# Discovery of functional NLRs using expression level, high-throughput transformation and large-scale phenotyping

**DOI:** 10.1038/s41477-025-02110-w

**Published:** 2025-09-23

**Authors:** Helen J. Brabham, Inmaculada Hernández-Pinzón, Chizu Yanagihara, Noriko Ishikawa, Toshiyuki Komori, Oadi N. Matny, Amelia Hubbard, Kamil Witek, Alexis Feist, Hironobu Numazawa, Phon Green, Antonín Dreiseitl, Naoki Takemori, Toshihiko Komari, Roger P. Freedman, Brian Steffenson, H. Peter van Esse, Matthew J. Moscou

**Affiliations:** 1 The Sainsbury Laboratory, University of East Anglia, Norwich, UK; 2 2Blades, Evanston, IL USA; 3 Plant Innovation Center, Japan Tobacco Inc., Iwata, Japan; 4 Agri-Bio Research Center, Kaneka Corporation, Iwata, Shizuoka Japan; 5 Department of Plant Pathology, University of Minnesota, St. Paul, MN USA; 6 NIAB, Cambridge, UK; 7 Department of Integrated Plant Protection, Agrotest Fyto Ltd, Kroměříž, Czech Republic; 8 Gatsby Charitable Foundation, London, UK; 9 Present Address: USDA-ARS, Cereal Disease Laboratory, University of Minnesota, St. Paul, MN USA

**Keywords:** Plant immunity, Plant biotechnology, Transgenic plants

## Abstract

Protecting crops from diseases is vital for the sustainable agricultural systems that are needed for food security. Introducing functional resistance genes to enhance the plant immune system is highly effective for disease resistance, but identifying new immune receptors is resource intensive. We observed that functional immune receptors of the nucleotide-binding domain leucine-rich repeat (NLR) class show a signature of high expression in uninfected plants across both monocot and dicot species. Here, by exploiting this signature combined with high-throughput transformation, we generated a wheat transgenic array of 995 NLRs from diverse grass species to identify new resistance genes for wheat. Confirming this proof of concept, we identified new resistance genes against the stem rust pathogen *Puccinia graminis* f. sp. *tritici* and the leaf rust pathogen *Puccinia triticina*, both major threats to wheat production. This pipeline facilitates the rapid identification of candidate NLRs and provides in planta gene validation of resistance. The accelerated discovery of new NLRs from a large gene pool of diverse and non-domesticated plant species will enhance the development of disease-resistant crops.

## Abstract (web summary)

Rapid discovery of functional resistance genes is enabled by a high-expression signature and high-throughput transformation. This approach identified 31 new resistance genes for wheat and can be applied across crops to support food security.

## Main

Protecting plant health is vital for building the sustainable food systems needed to end hunger and poverty, as plant diseases and pests cause major losses to crop yields worldwide^1,2^. New pathogen species and virulent strains can appear suddenly and spread rapidly, aided by globalization and climate change^3–7^, so there is an urgent need to accelerate methods for disease control. Using the plant immune system for defence is an effective method of crop protection. Plants contain immune receptors that recognize pathogen invasion, and a major class of intracellular plant disease resistance genes encode nucleotide-binding domain leucine-rich repeat (NLR) proteins^8^. Transferring NLRs within and between plant species has proved successful for disease resistance breeding; however, pathogens are constantly evolving and can overcome and evade existing NLRs used in the field. Introducing multiple NLRs in gene stacks can provide strong defence and limit this breakdown^9^, yet few NLRs are available in modern crop cultivars^10–12^. Wild relatives of crop species are a valuable source for new NLRs for disease resistance as they are often resistant to major agricultural pathogens^13,14^. Accessing the causal genes can be difficult due to limited availability of genetic resources; therefore, large-scale projects to characterize NLRs are required to find new resistances useful against a wide range of pathogens^11,12,15,16^.

NLRs recognize pathogen infection by directly interacting with pathogen molecules or via recognizing pathogen-induced modifications to plant host proteins^17,18^. Successful recognition results in defence responses to prevent the spread of infection, often including localized cell death^19^. The presence or altered regulation of some NLRs has been shown to cause deleterious effects: the presence of *Arabidopsis thaliana RPM1* reduced silique and seed production^20^, overexpression of *RPW8* (ref. ^21^) and *LAZ5* (ref. ^22^) can cause spontaneous cell death, and the lack of *PigmR* suppression in *Oryza sativa* causes a decrease in grain weight^23^. These observations, combined with the cell death function, resulted in the pervasive idea that NLRs require strict regulation to control defence responses^24–33^. NLRs were thought to be transcriptionally repressed across plants, but recent work has shown the cross-species transfer of NLRs without penalty^9,34^, and new knowledge of NLR function challenges these assumptions.

Here we found that multiple copies of the barley NLR *Mla7* are required for full complementation of resistance. This supports similar findings for *Mla3* (ref. ^35^), challenging the prevailing view that NLR expression must be maintained at a low level. We observed that an unexpectedly large number of NLRs are expressed in uninfected plants and that known functional NLRs are present among highly expressed NLR transcripts. We used this expression signature to predict functional NLR candidates at scale to find new resistance against two major diseases of wheat: stem rust caused by *Puccinia graminis* f. sp. *tritici* (*Pgt*) and leaf rust caused by *Puccinia triticina* (*Pt*). To date, 13 NLRs with efficacy against *Pgt* have been cloned^36–46^ and 7 against *Pt*^47–55^. We generated a transgenic array of 995 NLRs, using high-efficiency wheat transformation^56^, and identified 31 new resistant NLRs: 19 to stem rust and 12 to leaf rust. This proof-of-concept pipeline is applicable across plant species to rapidly identify new NLRs against various pathogens, enabling the development of disease-resistant crops.

## Results

### The NLR *Mla7* requires multiple copies to confer resistance to barley powdery mildew and wheat stripe rust

**Fig. 1:**
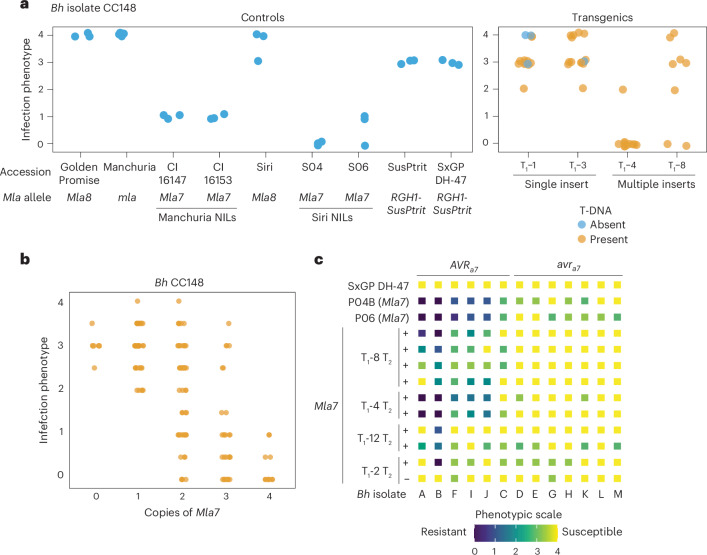
Multiple copies of *Mla7* are required to confer resistance to barley powdery mildew caused by *Bh.* **a**–**c**, Powdery-mildew-susceptible barley cv. SxGP DH-47 was transformed with *Mla7* driven by the *Mla6* promoter–5′UTR and the *Mla6* 3′UTR–terminator. Three single-copy insert lines (T_1_-1, T_1_-2 and T_1_-3) and three multiple-copy insert lines (T_1_-4, T_1_-8 and T_1_-12) were identified for *Mla7*. Resistance to *Bh* isolates carrying *AVR*_*a7*_ was observed in transgenic barley lines carrying multiple copies of *Mla7*. Specific recognition of the effector *AVR*_*a7*_ was retained across transgenic lines. Panel **a** shows infection phenotypes for the presence or absence of transfer DNA (T-DNA) for *Mla7* T_1_ families and controls inoculated with *Bh* isolate CC148. The presence and absence of T-DNA are shown in orange and blue, respectively. All phenotypes are on a scale from 0 to 4. Transparency and jittering were used to visualize multiple overlapping data points. As shown in **b**, multiple copies, not single copies, of *Mla7* under its native promoter/terminator are required to confer full resistance to *Bh* isolate CC148. Individual phenotypes from an F_2_ population with varying numbers of *Mla7* transgene copies derived from a cross of two single-insertion transgenic lines (T_1_-117 and T_1_-121) are plotted. As shown in **c**, multicopy lines carrying *Mla7* driven by the *Mla6* promoter/terminator confers race-specific resistance to barley powdery mildew (*Bh*). Controls include SxGP DH-47 and near-isogenic lines in the Pallas (*Mla8*) genetic background: P04B (*Mla7*) and P06 (*Mla7*). *Bh* isolates are ordered according to the presence (*AVR*_*a7*_) or absence (*avr*_*a7*_) of the effector recognized by *Mla7*. Isolates include 3-33 (A), Race I (B), X-4 (C), I-167 (D), K-200 (E), M-236 (F), Z-6 (G), C-132 (H), 120 (I), R86/1 (J), K-3 (K), KM18 (L) and MN-B (M). All experiments were performed twice with similar results; the data shown are the average of these experiments.

NLR resistance genes are thought to be maintained at low expression levels in uninfected plants to control defence responses. In barley (*Hordeum vulgare*), alleles of the NLR *Mla* are known to confer resistance to the barley powdery mildew pathogen *Blumeria hordei* (*Bh*). However, through transgene complementation, we observed that single insertions of *Mla7* driven by the *Mla6* promoter were insufficient to complement the resistance phenotype, whereas multicopy insertion lines expressed resistance to *Bh* isolate CC148 carrying the recognized effector *AVR*_*a7*_ (Fig. 1a). To mitigate the challenges associated with multisite insertion and transgene silencing, we developed single-copy transgenic lines expressing *Mla7* under its native promoter. We crossed two T_1_ families to develop an F_2_ population segregating for zero to four copies of *Mla7*. Higher-order copies were required for resistance to *Bh*, as only transgenic lines carrying two or more copies showed resistance to *Bh* isolate CC148 (*AVR*_*a7*_) (Fig. 1a), with full recapitulation of native *Mla7*-mediated resistance in lines with four copies (Fig. 1b). *Mla7* multicopy insert lines retained race specificity, as resistance was observed to only *Bh* isolates carrying *AVR*_*a7*_ (Fig. 1c). Although considerable variation was observed within and between transgenic families, increased copy number of *Mla7* does not cause auto-activity of resistance.

*Mla* alleles recognize multiple pathogens^35,57^ and in previous work, *Mla7* was shown to be in complete genetic coupling with *Rps7*, which confers resistance to *Puccinia striiformis* f. sp. *tritici* (*Pst*), indicating that they are probably the same gene^57^. Using the *Mla7* transgenic lines, we confirmed that *Mla7* also confers resistance to *Pst* (Supplementary Fig. 1a). In addition, as observed with *Bh*, multiple insertions of the *Mla7* transgene were required for *Pst* resistance (Supplementary Fig. 1b). Progeny of these multicopy lines showed unstable resistance, probably due to transgene silencing (Supplementary Fig. 2). Due to the correlation of copy number and phenotype, increased NLR expression is hypothesized to increase with higher copy number. *Mla7* natively exists with three identical copies in the haploid genome of barley cv. CI 16147 (Supplementary Fig. 3), supporting the hypothesis that a specific threshold of expression is required for function.

### Functional NLRs exhibit high steady-state expression levels

**Fig. 2:**
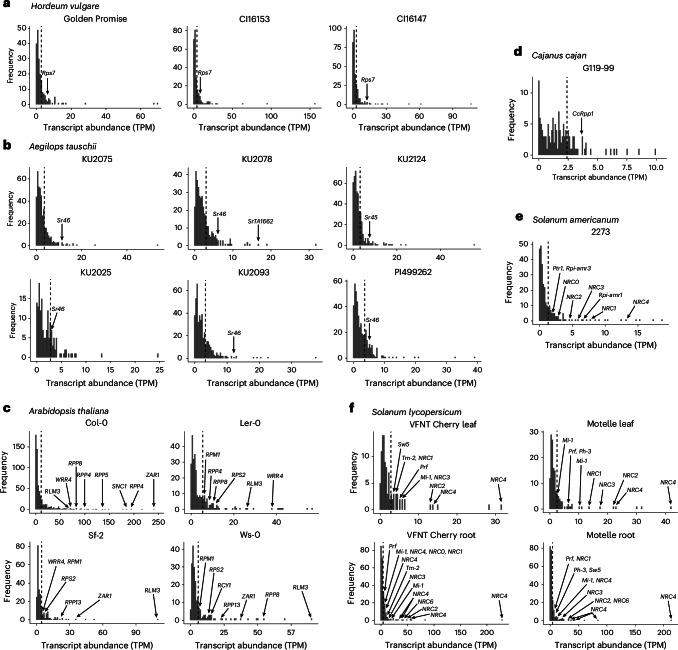
Functional NLRs are highly expressed in the de novo assembled transcriptome of unchallenged plants of *Aegilops*, *Cajanus*, *Arabidopsis*, *Solanum* and *Hordeum* species. **a**–**f**, Known functional NLRs from each species are among the most highly expressed NLR transcripts from unchallenged plant tissue (Supplementary Data 2). Transcript abundance was estimated from self-aligned RNA-seq data and measured in transcripts per million (TPM). Panel **a** shows transcript abundance of NLRs from the de novo assembled leaf transcriptomes of *Hordeum vulgare* accessions Golden Promise, CI16153 and CI16147. The expression of the functional resistance gene *Rps7* against *Bh* and *Pst* is indicated. Panel **b** shows transcript abundance of NLRs from the de novo assembled leaf transcriptomes of *Aegilops tauschii* accessions KU2025, KU2075, KU2078, KU2093, KU2124 and PI499262. The expression of the functional resistance genes *Sr45*, *Sr46* and *SrTA1662* against *Pgt* is indicated. Panel **c** shows transcript abundance of NLRs from the leaf transcriptomes of *Arabidopsis thaliana* accessions Col-0, Ler-0, Sf-2 and Ws-0. The expression of the functional resistance genes *RPP4*, *RPP5*, *RPP8* and *RPP13* to downy mildew (caused by *Hyaloperonospora arabidopsidis*); *WRR4* against white rust (*Albugo candida*); *ZAR1* against *Pseudomonas syringae* and *Xanthomonas campestris* pv. *campestris*; *RPS2* and *RPM1* against *Pseudomonas syringae*; *RCY1* against cucumber mosaic virus; and alleles of *RLM3* against grey mould (*Botrytis cinerea*), dark leaf spot of cabbage (*Alternaria brassicicola*) and dark spot of crucifers (*Alternaria brassicae*) is indicated. Panel **d** shows transcript abundance of NLRs from the *Cajanus cajan* accession G119-99 leaf transcriptome. The expression level of *CcRpp1*, which confers resistance to Asian soybean rust (*Phakopsora pachyrhizi*)^58^, is indicated. Panel **e** shows transcript abundance of NLRs from the de novo assembled leaf transcriptome of *Solanum americanum* accession SP2273. The expression of the functional resistance genes *Rpi-amr1* and *Rpi-amr3* to late blight (*Phytophthora infestans*) is indicated. An allele of *Ptr1*, which recognizes *Pseudomonas syringae* pv. *tomato* and *Ralstonia pseudosolanacearum* in *Solanum lycopersicoides*, is also shown. Helper NLRs are annotated as *NRC1*, *NRC2*, *NRC3*, *NRC4* and *NRC0* as defined by Kourelis et al.^140^. *NRC6* is not present in the dataset. Panel **f** shows transcript abundance of NLRs from *Solanum lycopersicum* cultivars VFNT Cherry and Motelle carrying *Mi-1* with resistance to root-knot nematodes (*Meloidogyne* spp.), the potato aphid (*Macrosiphum euphorbiae*) and the sweet potato whitefly (*Bemisia tabaci*). *Mi-1* is highly expressed in both cultivars in both leaf and root tissue. The expression levels of additional known functional NLRs *Tm-2* for resistance to tobamoviruses including tomato mosaic virus and tobacco mosaic virus, *Prf* for resistance to *Pseudomonas syringae* pv. *tomato*, *Sw5* for resistance to a broad range of viruses across paralogues and *Ph-3* for resistance to *P. infestans* are indicated. Helper NLRs are annotated as *NRC1*, *NRC2*, *NRC3*, *NRC4*, *NRC6* and *NRC0* as defined by Kourelis et al.^140^.

To investigate NLR expression, we assessed the expression levels of known characterized NLRs across six plant species of both monocots and dicots using sequencing data from uninfected leaf tissue. In monocots, the barley resistance genes *Rps7*/*Mla8* and *Rps7*/*Mla7* against *Bh* and *Pst* are present in highly expressed transcripts (Fig. 2a and Supplementary Data 1). The *Aegilops tauschii*-derived *Pgt* resistance genes *Sr46*, *SrTA1662* and *Sr45* are also present in highly expressed NLR transcripts across accessions (Fig. 2b). As a model species, the dicot *A. thaliana* contains a large complement of characterized functional NLRs, and highly expressed NLR transcripts are enriched with known genes (Fig. 2c). The most highly expressed NLR in ecotype Col-0 is *ZAR1*, and collectively across ecotypes, highly expressed NLRs provide resistance to diverse pathogen species (Fig. 2c and Supplementary Data 2). Using the de novo assembled transcriptome of accession Col-0, we found that known NLRs are significantly enriched in the top 15% of expressed NLR transcripts compared with the lower 85% (*χ*^2^ (1, *n* = 616) = 4.2979, *P* = 0.038). Using a non-redundant set of the highest-expressed transcript for each NLR, we found that the top 14% of expressed NLR transcripts are enriched for known NLRs (*χ*^2^ (1, *n* = 141) = 4.5767, *P* = 0.032). NLRs in the top 15% are in NLR classes containing coiled-coil, nucleotide-binding-site, leucine-rich-repeat and Toll/interleukin1 receptor domains without additional non-canonical domains (CNL, NL, TN, TNL and TNLT; Supplementary Figs. 4 and 5). Overall, the expression level of the most highly expressed NLR is above the median and mean expression levels for all genes in *A. thaliana* accession Col-0, confirming that NLRs are not transcriptionally repressed in uninfected plants (Supplementary Figs. 4 and 5).

NLRs previously identified via traditional methods and bioinformatic approaches, such as *CcRpp1* from *Cajanus cajan*^58^ and *Rpi-amr1* from *Solanum americanum*^59^, were also found to be present in highly expressed NLRs in the respective species (Fig. 2d,e). The tomato NLR *Mi-1* provides resistance to potato aphid and whitefly in foliar tissue and the root-knot nematode in the roots. We found that *Mi-1* is highly expressed in both the leaves and roots of the resistant cultivars Motelle and VFNT Cherry, alongside the additional characterized NLRs (Fig. 2f and Supplementary Data 2). *Rpi-amr1* and *Mi-1* are dependent on additional NLRs for function and are present in a wider network described in Solanaceae species^59,60^. NLRs that recognize pathogen products or host modifications directly are described as ‘sensor’ NLRs, and these partner with ‘helper’ NLRs that facilitate immune signalling^61^. Known helper NLRs, designated with the prefix NRC in the Solanaceae, are also highly expressed (Fig. 2e,f and Supplementary Data 2). In addition, many helper NLRs display tissue specificity; *NRC6* is highly expressed in the roots but not the leaves of tomato cvs. VFNT Cherry and Motelle, and *NRC0* is highly expressed in the roots of cv. VFNT Cherry but lowly expressed in the leaves of both cultivars and in the roots of cv. Motelle (Fig. 2f). These results therefore show the importance of investigating the appropriate plant tissue relevant for the pathogen and indicate the tissue specificity of resistance.

### The most highly expressed isoform of *Rpi-amr1* is the functional NLR

**Fig. 3:**
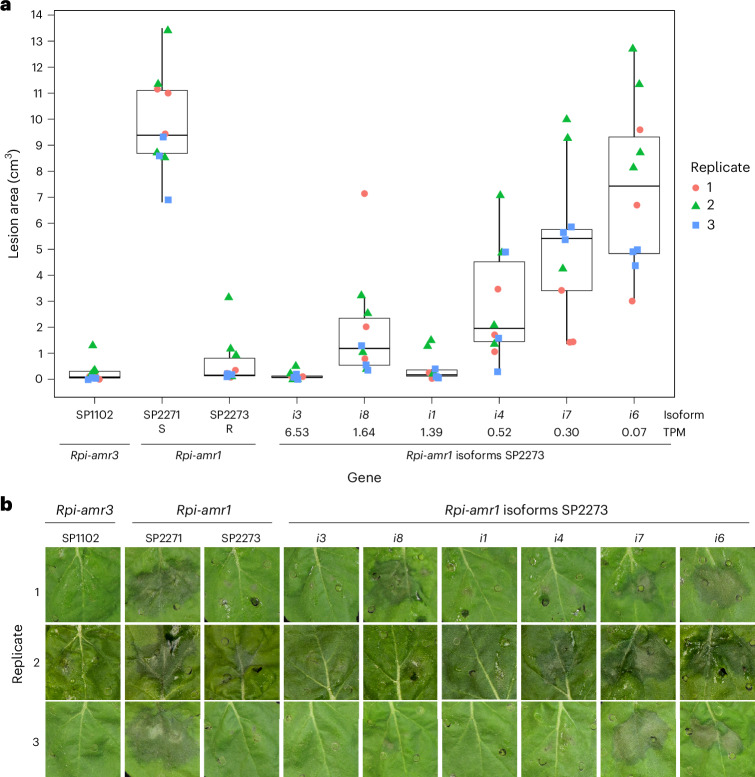
The most highly expressed isoform of *Rpi-amr1* provides resistance to *P. infestans.* **a**, Point plot and box plots of the lesion area of *P. infestans* infection in transient assays in *N. benthamiana* using different isoforms of *Rpi-amr1*. Individual biological replicates in each replicate are indicated with different coloured shapes (red circles for 1, green triangles for 2 and blue squares for 3). **b**, Representative photographs of lesions across replicates. Controls of resistant *Rpi-amr3* from accession SP1102, susceptible *Rpi-amr1* from accession SP2271 and resistant *Rpi-amr1* from accession SP2273 were included. *Rpi-amr1* isoforms from accession SP2273 are present in descending order of expression level: *i3* at 6.53 TPM, *i8* at 1.64 TPM, *i1* at 1.39 TPM, *i4* at 0.523343 TPM, *i7* at 0.30 TPM and *i6* at 0.07 TPM.

Multiple isoforms of each NLR are present in transcriptomes, and while the function of alternative splicing and isoform variation across NLRs is broadly uncharacterized, alternatively spliced variants of a few NLRs have been shown to modulate defence^62–65^. Different isoforms of *Rpi-amr1* are present in the assembled transcriptome of *S. americanum* accession SP2273 at varied expression levels. *Rpi-amr1* isoforms show sequence variation, including the presence/absence of the final exon (Supplementary Fig. 6). Expressing the different isoforms under the same *NRC4* promoter in transient assays in *Nicotiana benthamiana*, we found that the most abundant isoform, *i3*, provides resistance to *Phytophthora infestans* (Fig. 3). The isoform *i3* contains all exons as compared to the published sequence. Isoform *i1* also confers resistance to *P. infestans* as it contains all exons and is 99.3% similar to *i3*. Other isoforms present at lower expression levels in the transcriptome confer reduced levels of resistance to *P. infestans*. Transcript variants may be due to alternative splicing, the presence of paralogues or transcript assembly. As the most highly expressed isoform of *Rpi-amr1* is the functional transcript, this supports the idea that selecting the highest-expressed transcript is a feasible approach to select functional variants for other NLRs.

### Building an NLR array for *Pgt* resistance

**Fig. 4:**
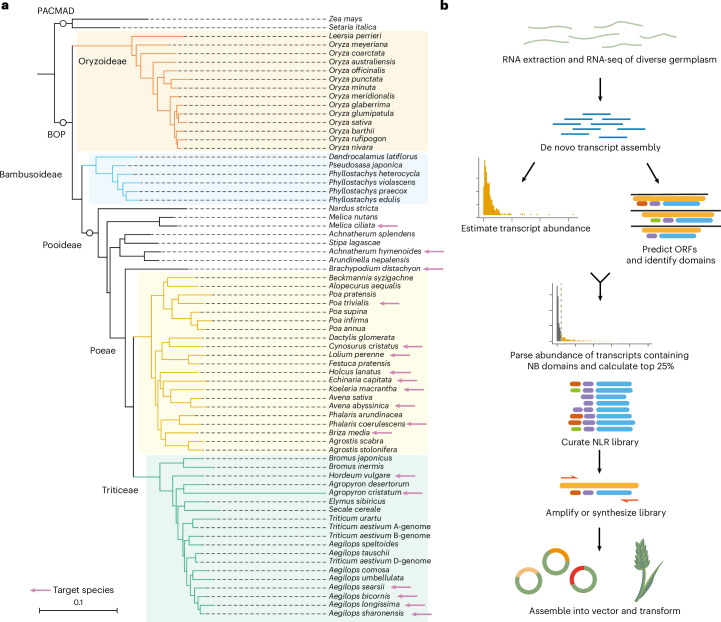
A pipeline for rapid identification of NLRs from diverse grass species. **a**, Phylogenetic tree of grass species in the PACMAD and BOP clades of the Poaceae. Species used for NLR discovery are indicated with pink arrows. **b**, The pipeline for the identification of highly expressed NLRs from RNA-seq data. The expression of transcripts containing a nucleotide-binding (NB) domain was used to identify the top 25% of expressed NLRs. Primers were designed on the open reading frame (ORF) of the NLRs, and the coding sequences were amplified via PCR and assembled into the transformation vector under the maize ubiquitin promoter.

We hypothesized that we could use the signature of higher expression levels in unchallenged tissue to mine functional NLRs from diverse plant germplasm. As a proof of concept, we sought to identify new NLRs against *Pgt* and *Pt*, which are major threats to global wheat production. NLRs providing resistance to rust have been previously characterized from close relatives of wheat^36,43,66^. We therefore investigated a total of 30 accessions of *Aegilops bicornis, A. longissima, A. searsii* and *A. sharonensis* to capture the genetic diversity present in this genus (Fig. 4a and Supplementary Table 1). To represent the breadth of diversity across the grasses, we included several species across the Triticeae and Poeae through to the distant wheat relatives spanning the Pooideae—many of which have not previously been assessed for rust resistance.

We identified highly expressed candidate functional NLRs from a total of 68 accessions of 18 plant species (Supplementary Table 1). We excluded NLRs from the MIC1 clade, as this clade is enriched with NLRs with integrated domains that require an additional NLR to function together as a pair^67–69^. The corresponding clade of paired helper NLRs was retained for the possibility of multi-sensor or interspecific NLR pairings. We chose the top 25% of NLR transcripts as this threshold encompasses known NLRs across plant species, particularly monocots (Fig. 2 and Supplementary Data 9). We selected the most highly expressed isoform for each NLR under the hypothesis that it is the functional variant. A total of 6,260 transformation events using high-efficiency *Agrobacterium*-mediated transformation of the wheat cultivar Fielder^56^ generated 5,177 independent T_1_ families for 995 NLR constructs driven by the maize ubiquitin promoter to create an array of transgenic wheat (Fig. 4b and Supplementary Data 3, 9 and 10). This array also includes the controls of known NLRs conferring stem rust resistance: *Sr33* (ref. ^39^), *Sr35* (ref. ^40^) and *Sr50* (ref. ^38^). *Lr21* was included as a control for leaf rust^55^.

### Identification of 19 NLRs conferring resistance to *Pgt*

**Fig. 5:**
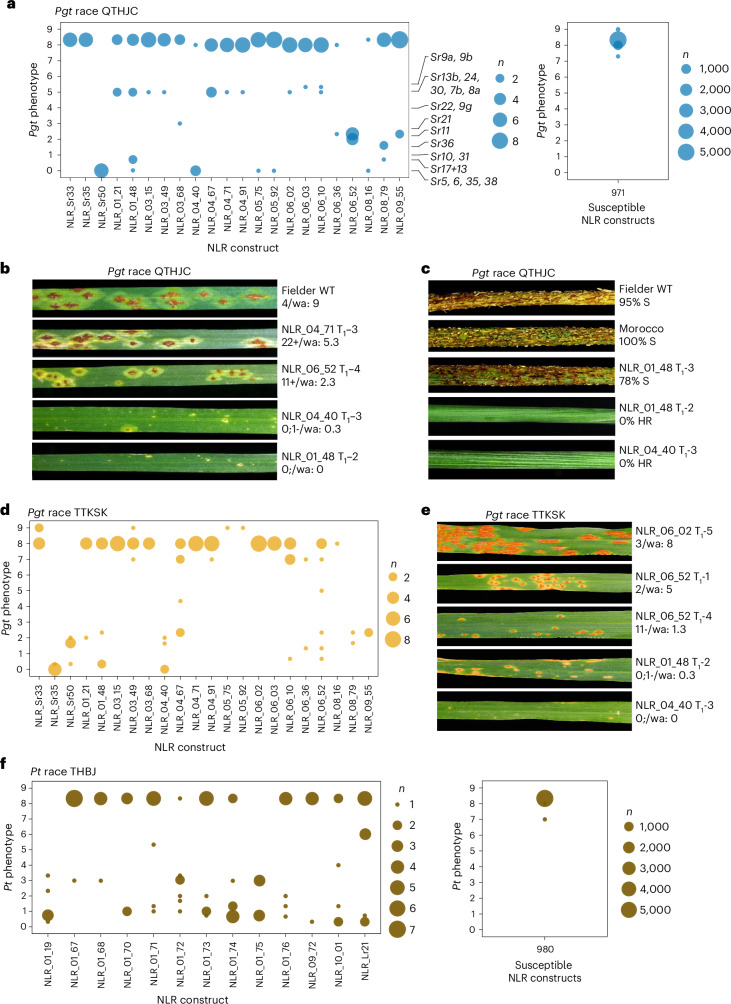
Identified NLRs provide resistance to *Pgt* races QTHJC and TTKSK and *Pt* race THBJ. **a**, A total of 19 NLRs conferred resistance against *Pgt* race QTHJC (Supplementary Data 4 and 5). Phenotypic scores from individuals in T_1_ families from each construct inoculated with *Pgt* race QTHJC are plotted on a weighted and transformed Stakman scale from completely resistant (0) to susceptible (9). Phenotypes of known *Sr* genes are indicated to the right of the plot (Supplementary Data 6). Circle size indicates the number of individuals with each phenotypic score. Individuals from the other 971 susceptible NLRs shown on the right were susceptible to *Pgt* and exhibited phenotypic scores similar to wild-type Fielder. **b**, Seedling leaves of T_2_ individuals from T_1_ families infected with *Pgt* race QTHJC under greenhouse conditions. An additional inoculation experiment was performed to obtain the photographs. From top to bottom: the wild-type (WT) Fielder susceptible control and four resistant individuals from resistant NLRs with the NLR construct and independent T_1_ family shown. The Stakman phenotype and corresponding weighted average (wa) per individual are also shown. S, susceptible; HR, highly resistant. **c**, Stem sections of selected individuals infected with *Pgt* race QTHJC under field conditions. From top to bottom: the wild-type Fielder susceptible control, a susceptible wheat control of cv. Morocco, a susceptible individual from a segregating family from NLR NLR_01_48 T_1_ family 3, a resistant individual from NLR NLR_01_48 T_1_ family 2 and a resistant individual from NLR NLR_04_40 T_1_ family 3. The NLR construct, the independent T_1_ family, and the phenotype of per cent severity and infection response are shown. S, susceptible; HR, highly resistant. **d**, Nine NLRs conferred resistance against *Pgt* race TTKSK. Individuals with resistance against *Pgt* race QTHJC were screened with *Pgt* race TTKSK. Phenotypes were scored on a weighted Stakman scale from highly resistant (0) to susceptible (9). Circle size indicates the number of individuals with each phenotypic score. Individuals from T_1_ families of NLRs NLR_01_21, NLR_01_48, NLR_04_40, NLR_04_67, NLR_06_10, NLR_06_36, NLR_06_52, NLR_08_79 and NLR_09_55 showed resistant phenotypes to *Pgt* race TTKSK. **e**, Seedling leaves of individuals from T_1_ families inoculated with *Pgt* race TTKSK under greenhouse conditions. From top to bottom: a susceptible individual of NLR NLR_06_02 independent T_1_ family 5 and four resistant individuals from resistant NLRs. The NLR construct, the independent T_1_ family, the Stakman phenotype and the corresponding weighted average per individual are shown. **f**, A total of 12 NLRs conferred resistance against *Pt* race THBJ (Supplementary Table 3 and Supplementary Data 8). Phenotypic scores from individuals in T_1_ families from each construct inoculated with *Pt* race THBJ are plotted on a weighted and transformed phenotypic scale from highly resistant (0) to susceptible (9). Circle size indicates the number of individuals with each phenotypic score. Individuals from the other 980 susceptible NLRs shown on the right were susceptible to *Pt* and exhibited phenotypic scores similar to that of wild-type Fielder.

We interrogated the transgenic wheat array for resistance to *Pgt* in greenhouse seedling assays using *Pgt* race QTHJC, a highly virulent isolate on wild-type Fielder (Fig. 5a,b, Supplementary Data 4 and Supplementary Table 2). A total of 19 NLRs provided resistance to *Pgt* race QTHJC when expressed in Fielder. Of these, resistance was observed in two or more independent T_1_ families for six NLRs and in one independent T_1_ family for 13 of the NLRs. Control lines carrying the known resistance gene *Sr50* were resistant to *Pgt* race QTHJC^9^ (Fig. 5a, Supplementary Data 4 and Supplementary Table 2). The resistant phenotypes observed were comparable to the differential wheat lines carrying known *Sr* resistance genes, which display phenotypes of 0; to 22+ (weighted averages 0 to 5.33) (Supplementary Data 6). All other remaining NLRs showed a susceptible phenotype (Fig. 5a, Supplementary Fig. 7 and Supplementary Data 4), including those carrying controls *Sr33* and *Sr35*. *Sr35* is ineffective against QTHJC as previously reported^40^, whereas the susceptible phenotype observed in *Sr33* transgenics may represent insufficient complementation by the transgene^70^.

Seed from individual resistant transgenic lines was bulked following the first round of inoculations, and T_2_ families were then tested to confirm efficacy in the field. A total of 17 of the 19 resistant NLRs were tested under field conditions, and four NLRs showed resistance to *Pgt* race QTHJC in the field (Fig. 5c, Supplementary Fig. 8 and Supplementary Data 5). To further validate resistance, we performed a secondary phenotypic screen using *Pgt* race QTHJC on all independent T_1_ transgenic lines per resistant NLR construct, as a random sub-sampling of independent lines was used in the primary screen. Resistance was observed in at least one T_1_ family for 16 of the resistant NLRs and in at least two T_1_ families for 10 of the NLRs (Supplementary Figs. 9 and 10, Supplementary Table 3 and Supplementary Data 7). Collectively across screens, resistance was observed in two or more T_1_ families for 14 of the resistant NLRs and in at least one T_1_ family for 19 of the NLRs. As all bulked T_2_ plants from the primary screen were used in the field, T_2_ lines of the collection that were bulked separately were screened to assess the heritability of resistance. Resistance was observed in two or more T_2_ families for 9 of the NLRs and in at least one T_2_ family for 13 NLRs. Reduced resistance at the T_2_ generation was observed for the controls, with one family of *Sr50* showing resistance and *Sr33* showing susceptibility (Supplementary Figs. 9 and 10, Supplementary Table 3 and Supplementary Data 7). Reduced resistance could be due to transgene silencing or insufficient expression for complementation of the phenotype, as these lines were bulked without phenotypic selection, indicating the importance of promoter optimization for sufficient NLR expression.

To assess the breadth of pathogen recognition, the 19 NLRs resistant to *Pgt* race QTHJC were further tested against the widely virulent *Pgt* race TTKSK. Nine NLRs also conferred resistance to *Pgt* race TTKSK. Ten NLRs exhibited race specificity, conferring resistance to *Pgt* race QTHJC and susceptibility to *Pgt* race TTKSK. The controls *Sr35* and *Sr50* showed resistance to TTKSK, and *Sr33* showed susceptibility (Fig. 5d,e, Supplementary Data 4 and Supplementary Table 2).

### Identification of 12 NLRs conferring resistance to *Pt*

The value of the transgenic array is the ability to repeatedly screen it against diverse pathogens. We further evaluated the array against *Pt*, the fungal wheat leaf rust pathogen. A total of 12 new NLRs provided resistance to *Pt* race THBJ in seedling glasshouse assays (Fig. 5f, Supplementary Fig. 7, Supplementary Data 8 and Supplementary Table 4). Of these, resistance was observed in two or more independent T_1_ families for eight NLRs and in one independent T_1_ family for four of the NLRs. The cloned leaf rust resistance gene, *Lr21* (ref. ^55^), was included as a resistant control and showed resistance to THBJ (Fig. 5f and Supplementary Fig. 10). Two NLRs identified in this screen matched known resistance genes: NLR_01_19, which matches *Yr87*/*Lr85* (*Aegilops sharonensis*), and NLR_10_01 which is *Mla37-1* (*Hordeum vulgare*)^52,71^. All transgenic lines carrying the NLRs resistant to *Pgt* were susceptible to *Pt*. NLRs within the transgenic array therefore retain pathogen recognition specificity, and the resistant phenotypes observed are not due to constitutive defence activation by the transgenes.

### NLR hits are present across diverse phylogenetic clades

We investigated the distribution of the functional NLR hits against *Pgt* and *Pt* using a previously annotated phylogenetic tree of NLRs from grass species^67^. We found that the NLR hits are present across different phylogenetic clades (Supplementary Tables 2 and 4 and Supplementary Fig. 11). Six *Pgt* hits are present in clade 17, which contains the known rust resistance genes *Lr10*, *Sr33* and *Sr35* (ref. ^67^). Two *Pgt* hits are present in clade 24, which contains the known leaf rust resistance genes *Lr1* and *Lr21*. Five *Pgt* hits are derived from clade 7 NLRs enriched with known paired NLRs with a helper function from the MIC1 clade^67^. For *Pt* hits, two NLRs are present in each of clades 7, 17, 19, 20, 22 and 24 (Supplementary Table 4). Several putative orthologous groups of NLRs in clade 7 confer rust resistance, such as NLR_03_49 (*H. lanatus*), NLR_06_52 (*Aegilops sharonensis*) and NLR_09_55 (*B. media*). *Aegilops*-derived orthologous NLRs include clade 7 NLR_04_67 (*Aegilops bicornis*) and NLR_06_36 (*Aegilops searsii*); one NLR group in clade 20 consisting of NLR_03_68 (*Aegilops sharonensis*) and NLR_06_10 (*Aegilops bicornis*); and different orthologues conferring resistance to wheat stem rust and wheat leaf rust in clade 22, NLR_01_74 (*H. lanatus*) and NLR_08_16 (*Aegilops* *sharonensis*).

## Discussion

Here we demonstrated that selecting highly expressed NLRs in uninfected plants facilitates rapid prediction of new NLR candidates from diverse germplasm. In our proof-of-concept study, the cross-species transfer of single NLRs—including NLRs from species never genetically investigated for resistance to wheat pathogens—provided 19 new resistance genes with efficacy against *Pgt*, including nine NLRs with resistance to the widely virulent *Pgt* race TTKSK (Ug99 lineage), and a further 12 against *Pt*. This method improves on previous cloning timelines, as 13 NLRs with efficacy against *Pgt*^36–45^ and 7 against *Pt*^47–55^ have been cloned to date. Variation in resistance was observed for the 19 NLRs across heritability, field efficacy and isolate specificity. Additional experimentation to investigate biological and/or technical mechanisms for this variation would further validate resistance. Among these new NLRs identified is the barley powdery mildew resistance gene *Mla37-1* (ref. ^71^), which we showed also confers resistance to wheat leaf rust, and the recently cloned *Yr87*/*Lr85* from *Aegilops sharonensis* independently identified through traditional genetic mapping and mutagenesis methods^52^. The majority of genes conferring resistance to wheat leaf rust in *Aegilops sharonensis* and the closely related species *Aegilops longissima* can be explained by *Yr87*/*Lr85*, as orthologues are present across accessions and confer the underlying resistance observed. This broad approach to curating NLR collections across species therefore minimizes redundancy and maximizes value to collect diverse NLRs. This NLR array is a valuable resource that can be repeatedly interrogated against other pests and diseases to validate new effective resistance genes in planta.

Further investigation is required to elucidate the mechanism of recognition of these new NLRs. While the association of NLR functionality and high expression occurs broadly across plant species, this may be non-uniform across different classes of NLRs and dependent on additional mechanisms. Several of the new *Pgt*- and *Pt*-resistant NLRs fall into phylogenetic clades with known rust resistance genes. Clade 17 contains the NLRs *Sr35* and *Mla*, which directly bind pathogen effectors^72,73^, and we hypothesize that clade 17 hits also function as single NLRs via direct recognition. Members of clade 7 are known to function as pairs, often with members of the MIC1 (C16) clade^67–69^. NLRs in pairs have distinct roles: NLRs in clade 16 are sensors that recognize pathogen effectors, and they require the clade 7 NLR helpers to activate immune signalling following recognition^18^. Here, the transfer of single clade 7 NLRs from divergent grass species provided resistance in wheat. These hits may represent a newly described function for helper NLRs in direct pathogen recognition, or they may be functioning with a new partner in the Fielder genetic background. If so, introducing new sensor NLRs or components of NLR pairs can expand or revive pathogen recognition with endogenous genes^74^.

In this study, no obvious macroscopic detrimental growth traits were observed across transgenic wheat lines in the greenhouse or field experiments. Moreover, transgenic lines were obtained for 99.6% of all transformed NLRs. Further testing is required to detect small differences in agro-morphological traits, but the absence of negative pleiotropic phenotypes is consistent with the cross-species transfer of NLRs into barley^34^ and a multi-transgene cassette transformed into wheat^9^. Further studies are required to elucidate the biological mechanisms of NLR expression, and subsequent work may be required for optimization; for example, NLRs in the array may need additional regulatory, promoter, terminator or intronic elements to function, so it is not possible to estimate the false-negative rate. Alternatively, these NLRs may provide resistance to other pathogens.

The concept that highly expressed NLRs may be detrimental was based on early observations of deleterious phenotypes^20,23–33,75–79^ and the induction of NLR expression following pathogen infection^29,80^. New understanding of NLR function can alleviate detrimental effects. For example, cell death or yield penalties caused by some NLRs can be suppressed via co-expression with their required partner or suppressor^18,23,68,81^. Other deleterious effects may be caused by mutations in the sequence or regulatory requirements of NLRs rather than expression levels. In *A. thaliana*, overexpression of the wild-type *SSI4* TIR-NLR sequence did not cause the stunting and cell death observed in the mutant variant, indicating that the phenotypes were caused by sequence mutation and not expression level^82^. Similarly, overexpression of *RPW8* alleles under the *35S* promoter showed no spontaneous cell death, whereas cell death was correlated with increased transgene copies of the genomic fragment carrying *RPW8* (ref. ^21^), indicating the disruption of gene regulation as causal^83^. The reduced height observed from induced mutations in *Rht13b* in wheat was independent of transgene copy number or gene expression^84^. Described examples of deleterious NLRs also involve guards or NLRs with additional integrated domains^20,85^. *RPM1* is lowly expressed in transcripts in *A. thaliana* Col-0, as are other known NLRs that guard host proteins such as *LOV1* (refs. ^20,86^). Different expression levels are observed across ecotypes for alleles, so effects may also be genotype dependent. In comparison, the expression of NLRs that directly recognize pathogen products may have less influence from host processes and proteins. Here we observed the requirement of high expression for *Mla7* function, and as other *Mla* alleles directly recognize effectors from *Bh*^73^, increased gene expression could compensate for any reduction in binding affinity towards their recognized effector under a positive dosage model^87^. Sufficient expression and protein abundance may also be required for NLR oligomerization and resistosome formation^72,88,89^. These results, alongside further advances in our understanding of NLR function, may explain the observation of deleterious phenotypes via mechanisms that are independent of NLR expression.

We have produced a high-throughput pipeline, designated NLRseek, which facilitates large-scale mining of resistance genes and provides access to genetic resistance in diverse plant species previously inaccessible to traditional methods. High-throughput phenotypic screening of plant transgenic arrays has been used successfully for identifying drought tolerance in rice^90^ and is a powerful tool that enables the study of pathogens less amenable to in vitro culture, such as shown here for *Pgt* and *Pt*. The value of this array is that it can be repeatedly screened with diverse pathogen species, isolates and races to validate NLRs directly in planta. The many diverse NLRs found using this approach would support the breadth of variation to deploy tailored gene stacks against major pathogens. This capability, combined with advances in gene cloning and genotype-independent transformation technology, ignites an exciting potential for the future of biotechnology to protect plant health and improve food security.

## Methods

### Native copy number variation of *Mla7*

Genomic DNA was extracted using a CTAB extraction approach from the Manchuria near-isogenic lines CI 16147 and CI 16153, which carry *Mla7* from two different donors (Multan and Long Glumes)^91^. Illumina sequencing was performed at Novogene (Cambridge, UK) using 150-bp paired-end reads. The reads were trimmed using Trimmomatic (v.0.39)^92^ with adapter clipping using TruSeq3-PE adapters and parameters 2:30:10, trimming of low-quality leading and trailing sequence with parameter 5, sliding window trimming with parameters 4:10 and a final minimum length of 36 bp. The copy numbers of *Mla7* and the control gene *Bpm* were determined using the *k*-mer analysis toolkit (KAT; v.2.4.1)^93^. The module sect was used with the default parameters to determine *k*-mer coverage over the gene sequences of *Mla7* and *Bpm*. R (v.4.1.2)^94^ and ggplot2 (v.3.3.6)^95^ were used to estimate copy number variation of *Mla7*.

### Transgenic complementation of *Mla7*

Promoter regions, UTRs and terminator regions of *Mla6* were amplified from barley CI 16151 (*Mla6*) and native genomic fragments of *Mla7* from CI 16153 (*Mla7*)^96^ using GoTaq Long PCR Master Mix (Promega). Constructs were developed as described in Bettgenhaeuser et al.^57^. Briefly, PCR fragments were assembled into the pBract202 binary vector (BRACT) via the Gibson reaction^97^. PCR fragments for assembly were produced with Phusion High-Fidelity DNA Polymerase (NEB) with 40-mer chimeric primers. Barley transformation was performed using the technique described in ref. ^98^ using the hygromycin resistance gene (*hyg*) as a selectable marker. Transformation was performed with the barley powdery mildew (*Bh*) and wheat stripe rust (*Pst*) susceptible line SusPtrit × Golden Promise DH-47 (SxGP DH-47)^99^. Copy number variation in transgenic plants was determined by quantitative real-time PCR using the selectable marker gene *hyg* (AttoDNA^100^). A population segregating for two T-DNA inserts was generated by crossing transgenic lines T_1_-117 and T_1_-121 (SxGP DH-47 transformed with *pMla7*::*Mla7*::*tMla7*) with the *Bh*- and *Pst*-susceptible accession Manchuria. T-DNA was mapped in segregating F_2_ populations to chromosomes 3H and 5H using SNP markers derived from the OPA markers 1_0702 and 2_1012 (ref. ^101^). F_2_ lines heterozygous for the T-DNA and homozygous at the *Mla* locus for the Manchuria allele (the non-functional *mla* allele) were crossed and validated in the resulting progeny for their allelic state at both the T-DNA and *Mla* loci. Selfed seed of this line was used for pathogen assays with *Bh* and *Pst*.

### Pathogen assays for *Mla7*

Pathogen assays with *Bh* and *Pst* were performed as described in Bettgenhaeuser et al.^57^. A collection of *Bh* isolates (*n* = 13) were selected from a collection containing 59 reference isolates collected in 12 countries in all non-polar continents over a period of 66 years (1953–2019) and maintained at the Agricultural Research Institute Kroměříž Ltd. Virulence patterns to 35 differential barley genotypes are shown in ref. ^102^. Prior to inoculation, purity was verified on standard barley lines^103^. *Bh* isolates were multiplied on leaf segments of the susceptible cultivar Bowman. *Bh* isolate CC148 was propagated on barley cv. Manchuria (CI 2330) prior to inoculation. Seedlings were placed horizontally, inoculated, rotated 180 °C after a resting period of 2 min for conidia to settle and then inoculated again. Susceptible controls in every experiment included Manchuria (CIho 2330), Pallas (CIho 11313), Siri (CIho 14846) and the barley cv. Siri-derived set of near-isogenic lines, each carrying a single mildew resistance gene^104^. *Bh* isolate CC148 assays were carried out in a negative-pressure containment greenhouse with supplemental lighting and temperature set at 18 °C day and 12 °C night. For pathogen assays using the diverse collection of *Bh* isolates, inoculation and evaluation protocols are described in detail in ref. ^105^. Briefly, seed was grown in a mildew-proof greenhouse under natural daylight. Central leaf segments of 15 mm were cut from fully expanded primary leaves after 14 days for each transgenic family and controls. The leaves were placed on water agar (0.8%) containing benzimidazole (40 mg l^−1^) in petri dishes with adaxial surfaces facing upwards. Leaf segments were placed at the bottom of a settling tower, and conidia from a fresh leaf segment of the susceptible cultivar were blown into the settling tower at a concentration of approximately 8 conidia per mm^2^. The petri dishes of leaf segments were incubated at 20 ± 2 °C under artificial light (cool-white fluorescent lamps providing 12 h light at 30 ± 5 μmol m^−2^ s^−1^). Infection responses were scored seven days after inoculation using a 0–4 scale where 0 indicates no visible mycelium or sporulation, and 4 indicates strong mycelial growth and sporulation^106^. Scoring was repeated a day later. Two replications were performed.

*Pst* inoculations were performed using a suspension of urediniospores in talcum powder at a weighted ratio of spores:talcum powder of 1:16 and applied to leaves using a spinning table. Plants were sealed and stored at 8 °C for 48 h immediately after inoculation. The plants were grown in a controlled-environment room under 16 h light/8 h dark. The phenotypes of the first leaves were scored 14 days post-inoculation using an incremental scale of 0 to 4 representing the surface area displaying an infection phenotype where 0 represented no chlorosis or no pustules of *Pst*, and 4 indicated infection across 100% of the surface area.

### Plant materials and growth conditions for RNA-seq analysis

Seeds of the grass species *Achnatherum hymenoides*, *Aegilops bicornis*, *Aegilops longissima*, *Aegilops searsii*, *Aegilops sharonensis*, *Agropyron cristatum*, *Avena abyssinica*, *Brachypodium distachyon*, *Briza media*, *Cynosurus cristatus*, *Echinaria capitata*, *Holcus lanatus*, *Hordeum vulgare*, *Koeleria macrantha*, *Lolium perenne*, *Melica ciliata*, *Phalaris coerulescens* and *Poa trivialis* were used for candidate NLR gene discovery.

Seeds were germinated on damp filter paper on petri dishes and placed at 4 °C for six to seven days to break seed dormancy. Germinated seeds were transferred into an in-house custom soil mix prepared by the horticultural services department at the John Innes Centre (JIC cereal mix: 65% peat, 25% loam, 10% grit, 3 kg m^−3^ dolomitic limestone, 1.3 kg m^−^^3^ PG mix and 3 kg m^−3^ Osmocote Exact). The seedlings were grown in a pest- and disease-free controlled-environment chamber under 16 h light at 20 °C/8 h dark at 16 °C. For RNA isolation, leaves of plants 12 to 35 days post germination were used depending on the species. The first and second leaves of plants were harvested for most plant species; however, the first to sixth leaves were used from species with smaller leaf sizes.

Seeds of *Solanum lycopersicum* cultivars VFNT Cherry (LA1221) and Motelle (LA2823) were obtained from the C.M. Rick Tomato Genetics Resource Center (https://tgrc.ucdavis.edu/). The seeds were germinated in an in-house custom soil mix prepared by the horticultural services department at the John Innes Centre (JIC multipurpose + grit: 90% peat, 10% grit, 4 kg m^−3^ dolomitic limestone, 0.75 kg m^−3^ PG mix and 1.5 kg m^−3^ Osmocote Bloom). Seedlings were grown in a pest- and disease-free controlled-environment chamber under 16 h light/8 h dark at 18 °C. Tissue was sampled for the RNA isolation after one month. Fully expanded leaves were used for leaf tissue, and the entire root system was used after washing in distilled water. For each tissue type, samples were pooled from three seedlings per cultivar.

For *Arabidopsis thaliana*, seeds of the lines Col-0, Ler-0, Mt-0 and Ws-0 were obtained from the Nottingham Arabidopsis Stock Centre (https://arabidopsis.info/). The seeds were surface sterilized in a sterilization chamber using chlorine gas for 5 h and sown on Murashige and Skoog media with 1% sucrose supplemented with 0.8% agar. The seeds were kept at 4 °C for two to three days and then grown at 22 °C under 16 h light. Seedlings were transferred to liquid Murashige and Skoog media with 1% sucrose in a 24-well tissue culture plate with seedlings in each well. The controlled-environment chamber used was a clean germination room free of plant pests and pathogens. The seedlings were sampled for RNA isolation nine to ten days post-germination.

### RNA extraction and sequencing

Total RNA was extracted from leaves of grass species, seedlings of *A. thaliana*, and leaf and root tissue of *S. lycopersicum* using a Trizol-phenol-based protocol according to the manufacturer’s instructions (Sigma-Aldrich; T9424). Barcoded Illumina TruSeq RNA HT libraries were constructed and pooled with four samples per lane on a single HiSeq 2500 lane run in Rapid Run mode using 150-bp paired-end reads. Reads were assessed for quality using FastQC (v.0.11.7)^107^ and trimmed before assembly using Trimmomatic (v.0.39) with the parameters set at ILLUMINACLIP, 2:30:10; LEADING, 5; TRAILING, 5; SLIDINGWINDOW, 4:15; and MINLEN, 36. De novo transcriptome assemblies were generated using Trinity^108^ with the default parameters (v.2013-11-10). kallisto (v.0.43.1)^109^ was used to estimate expression levels for all transcripts using the default parameters and 100 bootstraps.

### *Rpi-amr1* isoform characterization and *Phytophthora infestans* assays

Isoforms were identified from the transcriptome of *Solanum americanum* accession SP2273 using BLAST+ (v.2.2.31)^110^ of *Rpi-amr1* (GenBank: MW348763, NCBI). Sequence analysis was performed using Geneious Prime (v.2024.0.3) (https://www.geneious.com/features/prime). Gene isoforms were synthesized via Gene Universal (Supplementary Data 11). Coding sequences were expressed under the NRC4 promoter and terminator, and constructs were assembled using Golden Gate into the Level 1 acceptor pICH47732. Constructs of *Rpi-amr1* from the resistant accession SP2273, the susceptible accession SP2271 and *Rpi-amr3* from SP1102 (ref. ^59^) were used as controls. Transient complementation assays and *P. infestans* inoculation were performed as described previously^111,112^. Briefly, *Agrobacterium* liquid cultures were resuspended in MES buffer (10 mM MES, 10 mM MgCl_2_ and 150 mM acetosyringone) and adjusted to 0.3 OD_600_. A total of three to four leaves of different *N. benthamiana* plants were infiltrated with each construct per replicate with a total of three replicates. *P. infestans* 88069 was grown on rye media, and sporangia were harvested after ten days. Leaves were inoculated with two 10-μl droplets of a zoospore suspension (50,000 zoospores per ml). The inoculated leaves were incubated for 7 to 12 days on damp paper towels in a Sanyo cabinet at 16 °C under 16 h light and 8 h dark before the phenotypes were scored. The lesion area was measured from images using Fiji (ImageJ2, v.2.14.0/1.5f)^113^ and analysed using RStudio (v.2023.12.1+402)^114^.

### Identification of highly expressed NLRs

TransDecoder (v.4.1.0) LongOrfs^115^ was used to predict all open reading frames in de novo assembled transcriptomes. Transcript abundance was quantified using kallisto^109^. InterProScan (v.5.27-66.0)^116^ was used to annotate domains using Coils and the Pfam, Superfamily and ProSite databases. Any protein that contained both a nucleotide-binding domain and a leucine-rich repeat domain was retained. Histograms were generated using RStudio. The transcripts of known and characterized NLRs were identified from the transcriptome using a BLAST+ (v.2.2.31) search using the publicly available nucleotide sequence. Sequence similarity of the coding sequences was assessed using MUSCLE (v.5.1)^117^ using the default parameters.

### Building the NLR array and molecular cloning

Sequencing, de novo RNA-seq assembly, NLR identification and PCR primer development were completed for 81 accessions of 18 grass species. Of the 81 accessions sequenced, 68 accessions were progressed to molecular cloning including species in the genera *Achnatherum*, *Aegilops*, *Agropyron*, *Avena*, *Brachypodium*, *Briza*, *Cynosurus*, *Echinaria*, *Holcus*, *Hordeum*, *Koeleria*, *Lolium*, *Melica*, *Phalaris* and *Poa*.

Highly expressed NLRs were identified according to the pipeline described above, with the addition of Fast Approximate Tree Classification (FAT-CAT; https://github.com/shailen/FAT-CAT; ref. ^118^), which was used to classify nucleotide-binding domains on the basis of a phylogenetic tree developed from rice, *Brachypodium distachyon* and barley nucleotide-binding domains derived from NLRs^67^. NLR-encoding genes were advanced on the basis of the following requirements: the transcript must contain either a complete or 5′ partial open reading frame, the gene must be among the top 25% expressed NLRs and the gene must not belong to NLR families (C15/16) known to require an additional NLR. Among the candidate NLRs, redundancy was removed using CD-HIT (v.4.7)^119^ requiring 100% identity (*c* = 1.0).

For molecular cloning, PCR primers were developed using Gateway adapters attB1 and attB2 fused to the first 20 nucleotides of the start or end of the coding sequence, respectively. The proportion of cloned NLRs is variable according to species, guided by the available diversity of accessions in each species and the prevalence of resistance to target pathogens. PCR primers were developed for a total of 1,909 NLRs. In total, 1,019 NLRs were cloned into the Gateway pDONR entry vector. This set includes known resistance genes: *Sr33* (wheat stem rust^39^), *Sr35* (wheat stem rust^40^), *Sr50* (wheat stem rust^38^), *Lr21* (wheat leaf rust, Huang et al.^120^), *Yr10* (wheat stripe rust^121^), *Pm3b* (wheat powdery mildew^122,123^), *Mla3* (barley powdery mildew and rice blast^35^), *Mla7* (barley powdery mildew) and *Mla8/Rps7* (barley powdery mildew and wheat stripe rust^57,124^).

### Plant transformation

The NLRs in the entry clones were transferred to the destination binary vector pDEST2BL by the LR reaction of the Gateway system. NLRs were expressed under the maize ubiquitin promoter (Supplementary Data 10). The destination vector pDEST2BL includes the DsRed2 fluorescent protein for a visual selectable marker in the seed^125^. The resultant transformation vectors were introduced into *Agrobacterium tumefaciens* strain EHA105 by electroporation. *Agrobacterium* strains carrying the transformation vectors were used to transform wheat cv. Fielder^56^ with the modification that an immature embryo was cut into three pieces when transferred to the second selection medium. Fielder seeds were obtained from Kihara Institute for Biological Research, Yokohama City University. Briefly, 15 immature embryos were infected with each of the *Agrobacterium* strains, and up to seven independent events per NLR were grown to maturity. A total of 6,260 transformation events were achieved for 999 NLRs, and seed was obtained from T_1_ plants from 995 NLRs. Fifty or more seeds were obtained from 96.4% of the harvested events. A total of 5,177 T_1_ families were generated; the families were further subdivided on the basis of the fluorescence of the seed to a total of 10,646 DsRed2 groups.

### Inoculation and phenotyping with the wheat stem rust pathogen (*Pgt*) and the wheat leaf rust pathogen (*Pt*) at the seedling stage

Independent T_1_ families for each NLR construct were subsampled, and three seedlings from each T_1_ family were used for resistance phenotyping. Rust inoculations were performed according to the standard protocols used at the USDA-ARS Cereal Disease Laboratory and the University of Minnesota^126^. Briefly, on the day before inoculation, urediniospores of the rust pathogens were removed from a −80 °C freezer, heat-shocked in a 45 °C water bath for 15 min and then rehydrated in an 80% relative humidity chamber overnight. After germination rates were assessed^127^, 10 mg of urediniospores were placed into a gelatin capsule (size 00), and 700 ml of the light mineral oil (Soltrol 170, Chevron Phillips Chemical Company) carrier was added. The inoculum suspension was applied to 12-day-old plants (second leaf fully expanded) using custom atomizers (Tallgrass Solutions) pressured by a pump set at 25–30 kPa. Approximately 0.15 mg of urediniospores were applied per plant. Immediately after inoculation, the plants were placed in front of a small electric fan for 3–5 min to hasten the evaporation of the oil carrier from the leaf surfaces. The plants were off-gassed for an additional 90 min before being placed inside mist chambers. In the mist chambers, ultrasonic humidifiers (Vick’s model V5100NSJUV; Proctor & Gamble) were run continuously for 30 min to provide sufficient initial moisture for the germination of urediniospores. For the next 16–20 h, the plants were kept in the dark, and the humidifiers were run for 2 min every 15 min to maintain moisture on the plants. Light (400-W high-pressure sodium lamps emitting 300 mmol photons per s per m^2^) was provided for 2 to 4 h after the dark period. The chamber doors were opened halfway to allow the leaf surfaces to dry completely before the plants were returned to the greenhouse under the same conditions as described above^126^.

All rust phenotyping experiments were conducted in a completely randomized design. Accessions exhibiting variable reactions across experiments were repeated in an additional experiment if sufficient seeds were available. Stem rust infection types on the accessions were scored 12 days after inoculation using a 0 to 4 Stakman scale (0, 1−, 1, 1+, 2−, 2, 2+, 3−, 3, 3+, and 4)^128,129^. The semicolon symbol ‘;’ indicates a hypersensitive fleck. Raw seedling infection type data were converted to a numerical 0–9 linear scale as detailed in ref. ^130^. The initial phenotyping of transgenic lines was performed with the Minnesota, USA, *Pgt* race QTHJC (isolate 69MN399, originally collected in Minnesota, USA, and provided by Y. Jin, USDA-ARS Cereal Disease Laboratory, St. Paul, MN, USA). Lines found resistant to *Pgt* race QTHJC were further phenotyped against the more broadly virulent *Pgt* race TTKSK (isolate 04KEN156/04 provided by Y. Jin) from Kenya. Both races are virulent on the wheat cultivar Fielder. T_2_ plants were used for photographs of *Pgt* race QTHJC infection and for NLRs NLR_05_75, NLR_05_92, NLR_08_16, NLR_08_79 and NLR_09_55 for *Pgt* race TTKSK screening due to seed availability.

Wheat leaf rust screening was carried out using a protocol similar to that used for wheat stem rust screening but without the light period being provided at the end of the infection period^126^. *Pt* race THBJ (isolate 99ND588DLL, provided by J. Kolmer, USDA-ARS Cereal Disease Laboratory, St. Paul, MN, USA) was used for the assays. Leaf rust infection types were scored after inoculation using a 0 to 4 scale^131^, and the data were converted to a numerical 0–9 linear scale as described above.

### Inoculation and phenotyping with the wheat stem rust pathogen (*Pgt*) at the adult stage

Individual lines found resistant in the seedling assays with *Pgt* race QTHJC were bulked, and T_2_ families from multi-transgene lines and control lines were planted at the University of Minnesota Rosemount Research and Outreach Center in Rosemount, MN, in 2021 and 2022. The wheat cultivars Fielder, Morocco and LMPG-6 were used as susceptible controls. Field inoculations were performed with *Pgt* race QTHJC and as previously described in Luo et al.^9^. Several of the lines found resistant in the seedling stage were not available for testing in the field due to low seed production, and therefore partially overlapping field trials were used. Approximately 25 seeds per line were planted in each plot. When the first nodes of plants were detectable (31 Zadoks scale^132^), they were inoculated with a suspension of *Pgt* urediniospores (1 g urediniospores per 1 l Soltrol 170 mineral oil) using an ultra-low-volume sprayer (Mini-ULVA, Micron Group). Three additional inoculations were made in successive weeks to ensure high infection levels during the later stages of crop development. Severity was recorded as the visual percentage (0–100%) of stem and leaf sheath tissue covered by uredinia. Ratings were assessed using the modified Cobb scale^133^. The infection responses were recorded as highly resistant (clear hypersensitive infection sites but with no pathogen sporulation), resistant (minute to small uredinia surrounded by chlorosis or necrosis), moderately resistant (medium-sized uredinia often surrounded by chlorosis), moderately susceptible (medium to large erumpent uredinia with little or no chlorosis) or susceptible (very large erumpent uredinia with little or no chlorosis).

### Phylogenetic analyses

Phylogenetic analysis of the nucleotide-binding domains of NLRs was carried out as described by Bailey et al.^67^ using updated NLR gene annotations for barley^134^, *B. distachyon* (v.3.1; NCBI PRJNA32607 and PRJNA74771) and wheat^135^. Nucleotide-binding domains from NLRs were identified using HMMer (v.3.3.2) (http://hmmer.org/) hmmalign with the hidden Markov model encompassing the NB-ARC1-ARC2 domains^67^. The alignment was converted to an aligned FASTA file using esl-reformat and processed using the QKphylogeny script QKphylogeny_alignment_analysis.py with the parameters *d* = 0.3 (non-redundant), *b* = 0.5 (breadth coverage of alignment greater than or equal to 50%) and *d* = 0.3 (depth of coverage at each residue of greater than or equal to 30%). The phylogenetic tree was constructed using RAxML (v.8.2.12)^136^ using the PROTGAMMAJTT model and 1,000 bootstraps and visualized using iTOL (https://itol.embl.de/).

The Pooideae species phylogenetic tree was generated using the QKbusco pipeline (https://github.com/matthewmoscou/QKbusco). BUSCO (v.3.0.2)^137^ with the default parameters and the embryophyte_odb9 library was used to identify genes using annotated coding sequences (sequenced genomes) or open reading frames predicted using TransDecoder (v.4.1.0) from de novo assemblies (transcriptomes). QKbusco_merge.py was used to parse the BUSCO output and prepare FASTA files for multiple sequence alignment. The parameter status was set to fragmented to allow fragmented coding sequences to be included in the analysis. Codon-based multiple sequence alignment of individual genes was performed using PRANK (v.170427)^138^. Individual gene multiple sequence alignments were merged using QKbusco_phylogeny.py using a coverage depth of 40% at individual sites for inclusion in the alignment. The maximum likelihood phylogenetic tree was generated using RAxML (v.8.2.12) with the GTRGAMMA model.

### Reporting summary

Further information on research design is available in the Nature Portfolio Reporting Summary linked to this article.

## Supplementary information

Supplementary Information Supplementary Figs. 1–11 and Tables 1–4.

Reporting Summary

Supplementary Data 1 Additional references for known NLR genes.

Supplementary Data 2 Expression of NLRs from the transcriptomes of *Arabidopsis* and *Solanum* species.

Supplementary Data 3 Details of the NLRs present in the NLRseek transgenic wheat collection.

Supplementary Data 4 Details on the NLRseek transgenic lines and their raw infection types, linearized infection type scores and weighted averages to races QTHJC and TTKSK of the stem rust pathogen (*Pgt*) at the seedling stage in the greenhouse.

Supplementary Data 5 Details on the NLRseek transgenic lines and their rust severity and infection responses to race QTHJC of the stem rust pathogen (*Pgt*) in field trials conducted in Rosemount, Minnesota, in 2021 and 2022.

Supplementary Data 6 Detailed infection type and weighted average phenotypes of known stem-rust-resistant *Sr* genes.

Supplementary Data 7 Details on the secondary NLRseek transgenic lines and their raw infection types including previous screening tests for comparison, linearized infection type scores and weighted averages to races QTHJC and TTKSK of the stem rust pathogen (*Pgt*) at the seedling stage in the greenhouse across T_1_ and T_2_.

Supplementary Data 8 Details on the NLRseek transgenic lines and their raw infection types, linearized infection type scores and weighted averages to race THBJ of the leaf rust pathogen (*Pt*) at the seedling stage in the greenhouse.

Supplementary Data 9 Details of the NLRs identified from donor species and transformed as part of the NLRseek collection.

Supplementary Data 10 Sequence of the maize ubiquitin promoter used in construct development.

Supplementary Data 11 Sequences of the synthesized *Rpi-amr1* isoforms.

## Data Availability

The whole-genome sequencing data from barley accessions CI 16147 and CI 16153 have been deposited in NCBI under BioProject PRJNA952654. The RNA-seq data for *Arabidopsis thaliana*, tomato and diverse Pooideae species have been deposited in NCBI under BioProjects PRJNA928100, PRJNA927036 and PRJNA913397, respectively. The GenBank identifiers for the transformation construct sequence for *Mla7* under the *Mla6* promoter/terminator and the native sequence are MZ555770 and OQ859100, respectively. The databases used for protein domain analysis include Pfam, Superfamily and ProSite. The raw data and uncropped images are available via figshare at 10.6084/m9.figshare.28680800.v1 (ref. ^139^).

## Code availability

The scripts used for data analysis and figure preparation are available via figshare at 10.6084/m9.figshare.28680800.v1 (ref. ^139^).
