# Supplementary material for: Discovery of functional NLRs using expression level, high-throughput transformation and large-scale phenotyping
Source: Nat Plants. 2025 Sep 23;11(10):2100–14. doi: 10.1038/s41477-025-02110-w (PMC12537499; doi:10.1038/s41477-025-02110-w)
Supplement: Supplementary file 1 — Supplementary Figs. 1–11 and Tables 1–4. [file 41477_2025_2110_MOESM1_ESM.pdf]

nature plants

Supplementary information https://doi.org/10.1038/s41477-025-02110-w

Discovery of functional NLRs using expression level, high-throughput transformation and large-scale phenotyping

In the format provided by the authors and unedited

Supplemental:

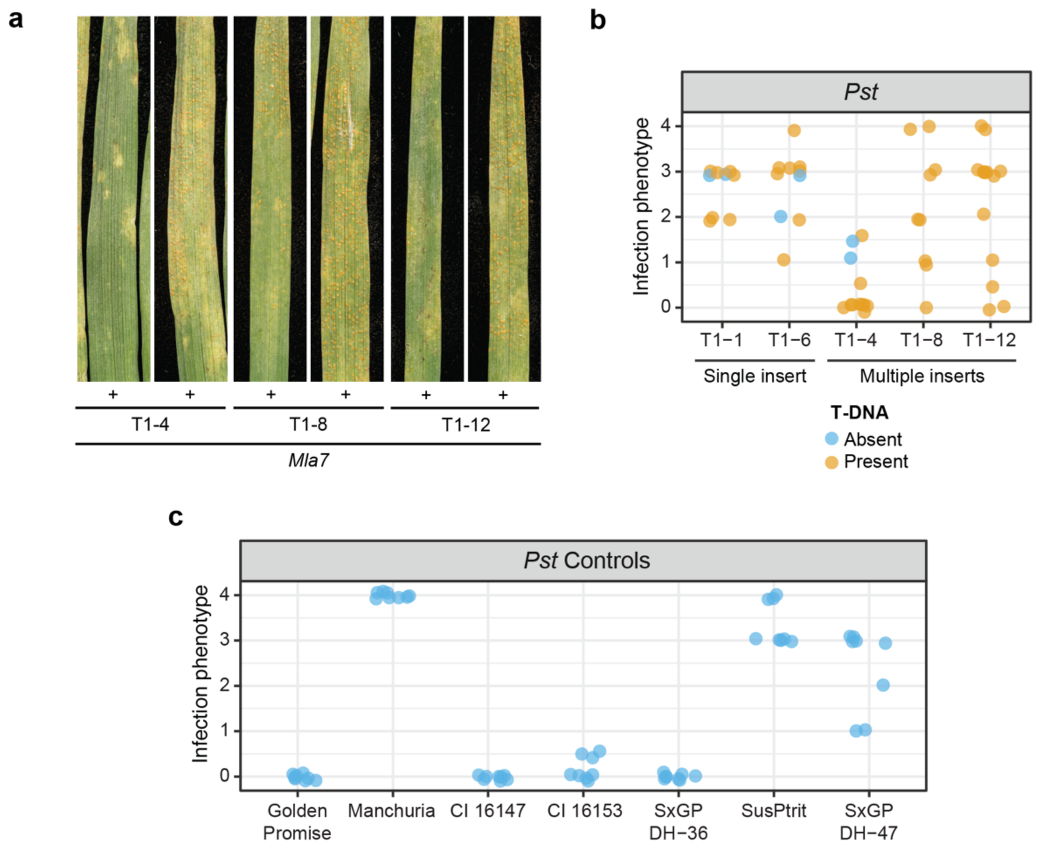

Supplemental Figure 1. *Mla7* confers resistance to wheat stripe rust (*P. striiformis* f. sp. *tritici*) isolate 16/035. Wheat stripe rust susceptible barley cv. SxGP DH-47 was transformed with *Mla7* driven by the *Mla6* promoter and *Mla6* terminator. Two single copy insert lines (T_1-1 and T_1-6) and three multiple copy insert lines (T_1-4, T_1-8, and T_1-12) were identified for *Mla7*. **A)** Resistance to wheat stripe rust isolate 16/035 was observed in transgenic barley lines carrying *Mla7*. **B)** Multiple T-DNA insertions are required to recapitulate wild-type resistance to *Pst*, as only transgenic lines carrying more copies of *Mla7* displayed resistant phenotypes of 0. Infection phenotypes for individual leaves in T_1 families. Presence or absence of T-DNA is shown in orange and blue, respectively. All phenotypes are on a scale from 0 to 4 representing the degree of leaf area covered with uredinia. Transparency and jittering were used to visualize multiple overlapping data points. **C)** Resistant control barley lines for *Pst* inoculations included Golden Promise (*Mla8*/*Rps7*), CI 16147 (*Mla7*/*Rps7*), CI 16153 (*Mla7*/*Rps7*), and SxGP DH-36 (*Mla8*/*Rps7*). Susceptible controls included Manchuria, SusPtrit, and SxGP DH-47 the genetic background used for *Mla7* transgenic lines. The experiment was carried out in parallel with results and controls of Bettgenhaeuser *et al.* (2021).

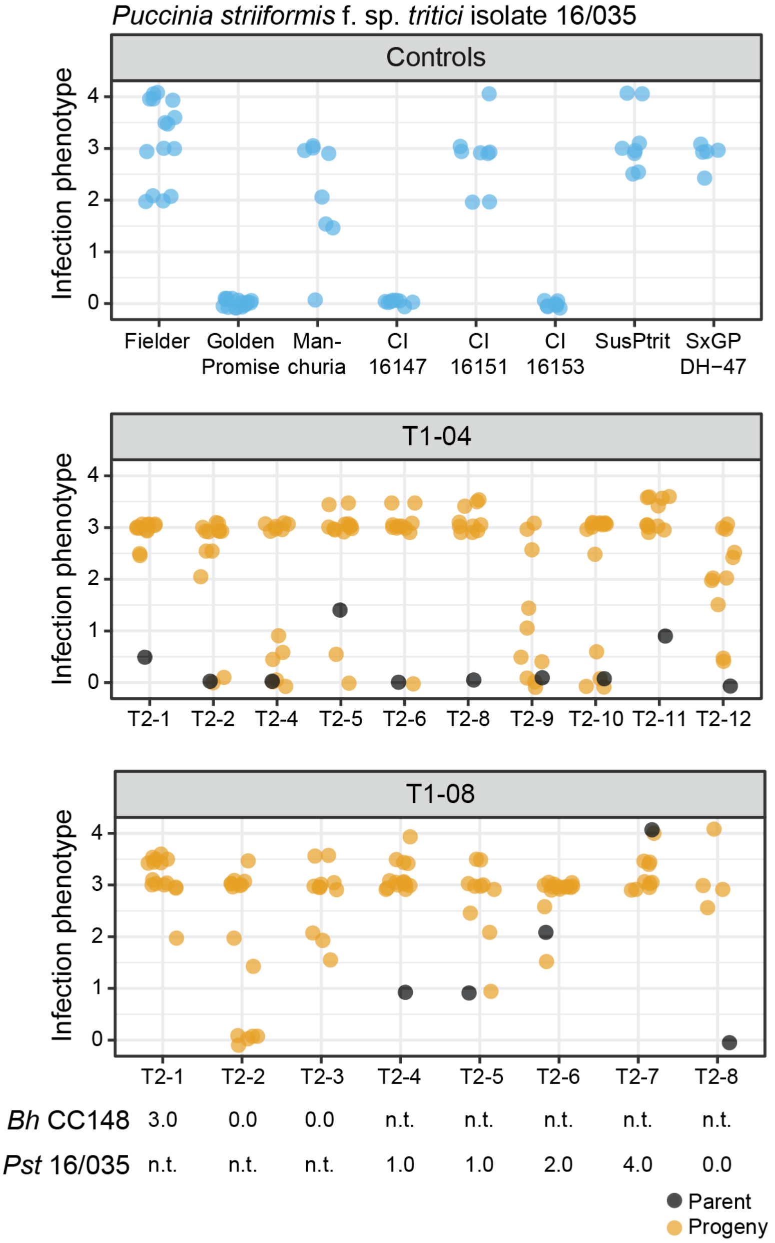

Supplemental Figure 2. *Mla7* transgenics show variation in heritability of resistance to wheat stripe rust (*P. striiformis* f. sp. *tritici; Pst*) isolate 16/035 from the T_1 to T_2 generations. Wheat stripe rust susceptible barley cv. SxGP DH-47 was transformed with *Mla7* driven by the *Mla6* promoter and *Mla6* terminator. Two multiple copy insert lines (T_1-4 and T_1-8) expressing resistance to barley powdery mildew (*Bh* CC148) and wheat stripe rust were tested for resistance in the T_2 generation. Phenotype of the control lines to *Pst* isolate 16/035 shown with blue circles, T_1 parent with black circles, and the T_2 progeny with orange circles. Resistant control barley lines for *Pst* inoculations included Golden Promise (*Mla8*/*Rps7*), CI 16147 (*Mla7*/*Rps7*), and CI 16153 (*Mla7*/*Rps7*). Susceptible controls included Manchuria, SusPtrit, SxGP DH-47 the genetic background used for *Mla7* transgenic lines, and the wheat line Fielder.

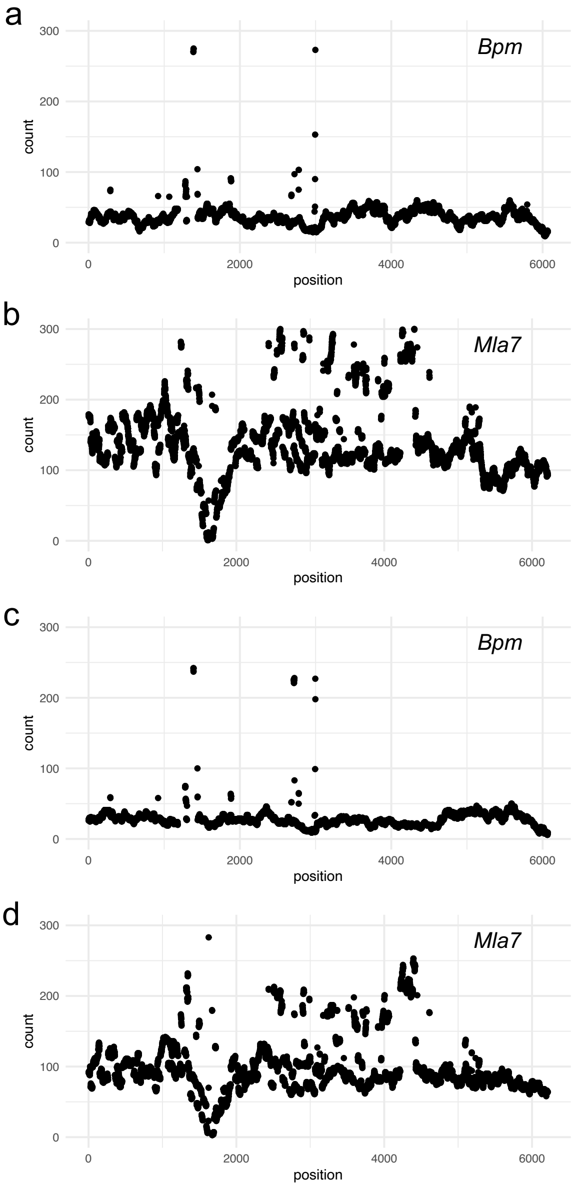

Supplemental Figure 3. Barley accessions CI 16147 and CI 16153 carry three copies of *Mla7*. Whole genome sequencing was performed on CI 16147 and CI 16153 and k-mer analysis for the genes *Bpm* and *Mla7*. **A**) Coverage analysis of *Bpm* in CI 16147 estimates a median coverage of 36 (1 copy). **B**) Coverage analysis of *Mla7* in CI 16147 estimates a median coverage of 117 (~3.25 copies). **C**) Coverage analysis of *Bpm* in CI 16153 estimates a median coverage of 27 (1 copy). **D**) Coverage analysis of *Mla7* in CI 16153 estimates a median coverage of 87 (~3.22 copies).

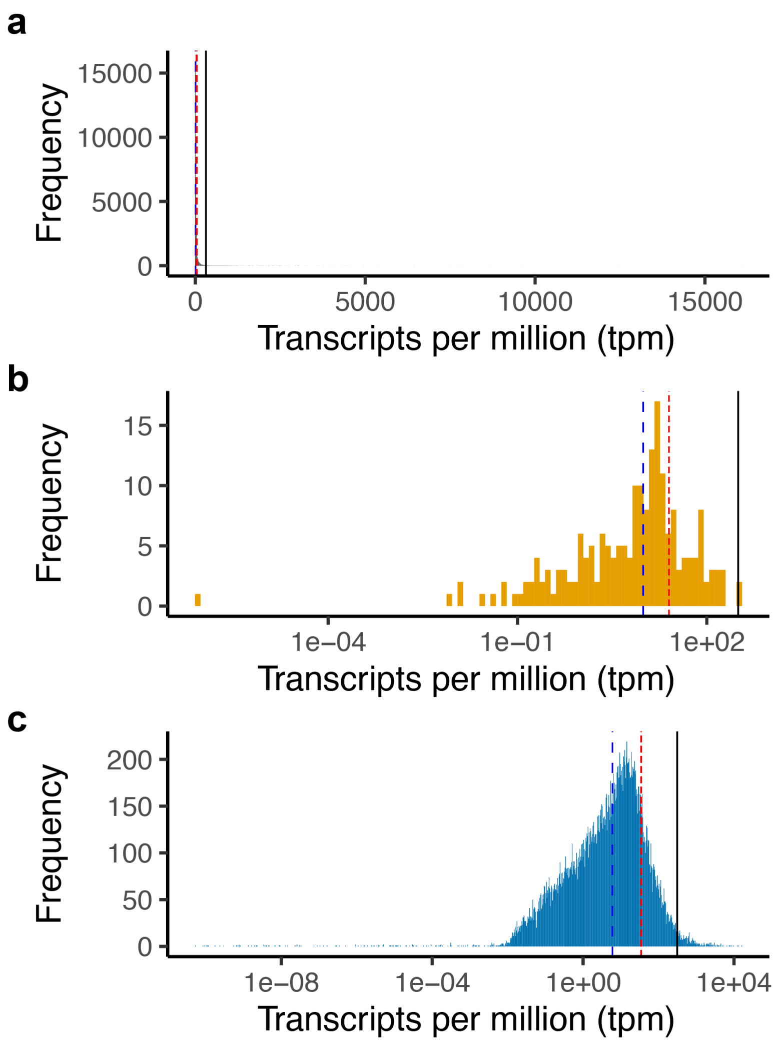

Supplemental Figure 4. Expression level of all genes in *A. thaliana* ecotype Col-0. Median gene expression level of 5.98 tpm annotated with a blue dashed line. Mean gene expression level of 34.60 tpm annotated with a shorter dashed red line. The expression level of the highest expressed NLR at 313.68 tpm indicated with a solid black line. An average of the publicly available datasets of replicates SRR5197904, SRR5197905, and SRR5197906 used for analysis. TAIR10 gene annotations and NLR annotations from Meyers *et al.*, (2003) were used. **A)** Histogram of untransformed gene expression levels. **B)** Histogram of gene expression of all NLR genes plotted on a $\log_{10}$ transformed scale of transcripts per million. **C)** Histogram of gene expression of all non-NLR genes plotted on a $\log_{10}$ transformed scale of transcripts per million.

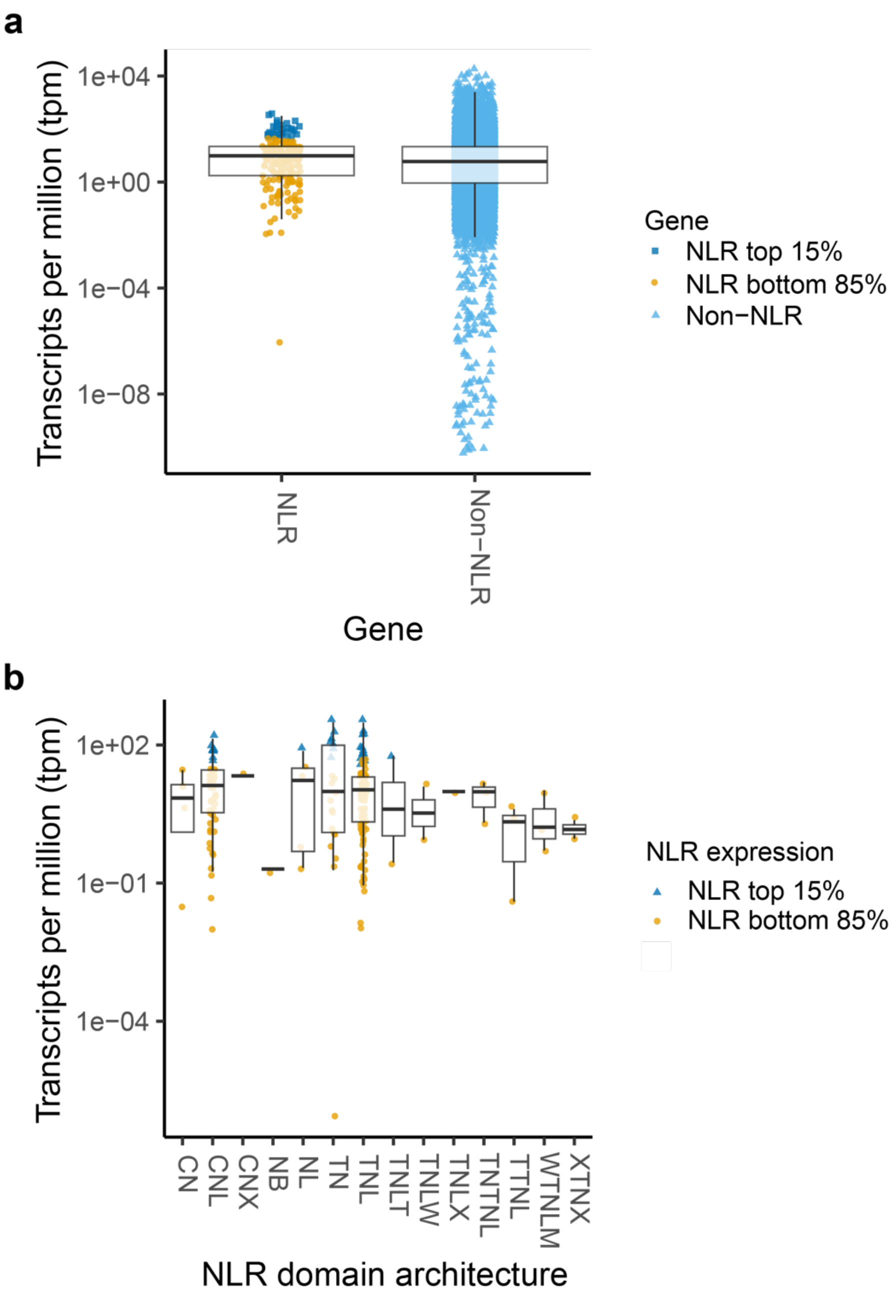

Supplemental Figure 5. Comparative NLR expression in *A. thaliana* ecotype Col-0. Box plots of gene expression from *A. thaliana* ecotype Col-0. An average of the publicly available datasets of replicates SRR5197904, SRR5197905, and SRR5197906 used for analysis. Median gene expression indicated by the thicker horizontal line within the white box denoting the interquartile range. **A)** Expression level of NLRs compared to non-NLRs plotted on a $\log_{10}$ transformed scale of transcripts per million. The top 15% of expressed NLRs indicated with dark blue squares, the bottom 85% of expressed NLRs indicated with orange circles, and non-NLRs indicated with light blue triangles. **B)** Expression level of different NLR domain classes as classified in Meyers *et al.*, (2003, 2002) plotted on a $\log_{10}$ transformed scale of transcripts per million. NLR domain architecture indicated as: CN=Coiled coil (CC)-nucleotide binding

site (NBS), CNL = CC-NBS-leucine rich repeat (LRR), CNX = CC-NBS-X (where X indicates additional variable domains), NB = NBS, NL = NBS-LRR, TN = TIR-NBS, TNL = TIR-NBS-LRR, TNLT = TIR-NBS-LRR-TIR, TNLW = TIR-NBS-WRKY, TNLX = TIR-NBS-LRR-X, TNTNL = TIR-NBS-TIR-NBS-LRR, TTNL = TIR-TIR-NBS-LRR, WTNLM = WRKY-TIR-NBS-LRR-Kinase like domain, XTNX = X-TIR-NBS-X. The top 25% of expressed NLRs indicated with blue triangles and the bottom 75% of expressed NLRs indicated with orange circles.

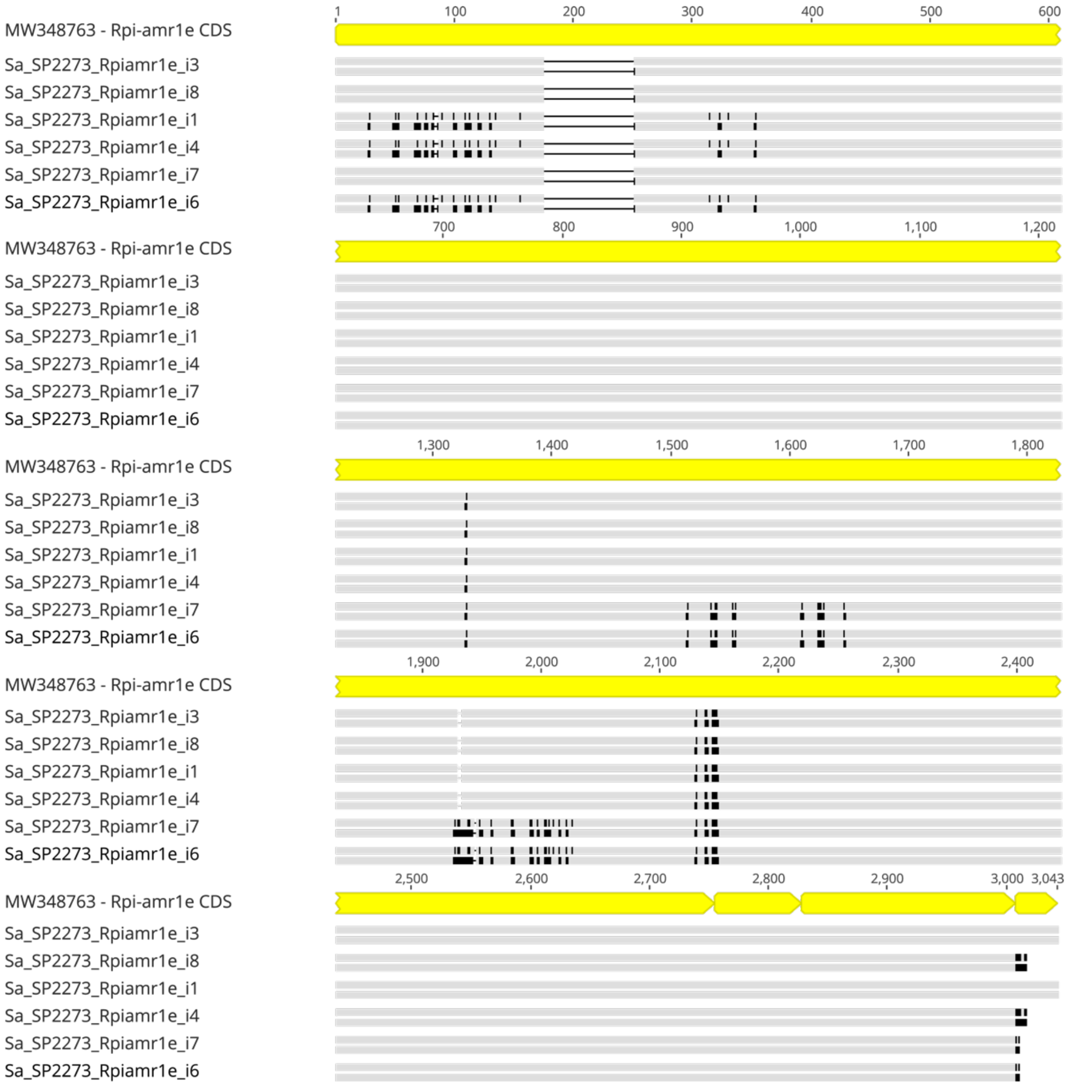

Supplemental Figure 6. Sequence alignment of *Rpi-amr1* isoforms. Alignment of isoforms of *Rpi-amr1* identified from the *de novo* assembled transcriptome of *S. americanum* accession SP2273 against the *Rpi-amr1* publicly available sequence (GenBank: MW348763). The exon structure of *Rpi-amr1* (GenBank: MW348763) indicated in the yellow annotations. *Rpi-amr1* isoforms listed in descending order of expression level, with isoform *i3* being the most highly expressed. For each isoform, the top grey bar shows nucleotide sequence similarity, and the lower grey bar shows amino acid sequence similarity. Black bars indicate sequence differences and the black line with the break in the grey bar indicates presence/absence variation. Multiple sequence alignment performed using Geneious.

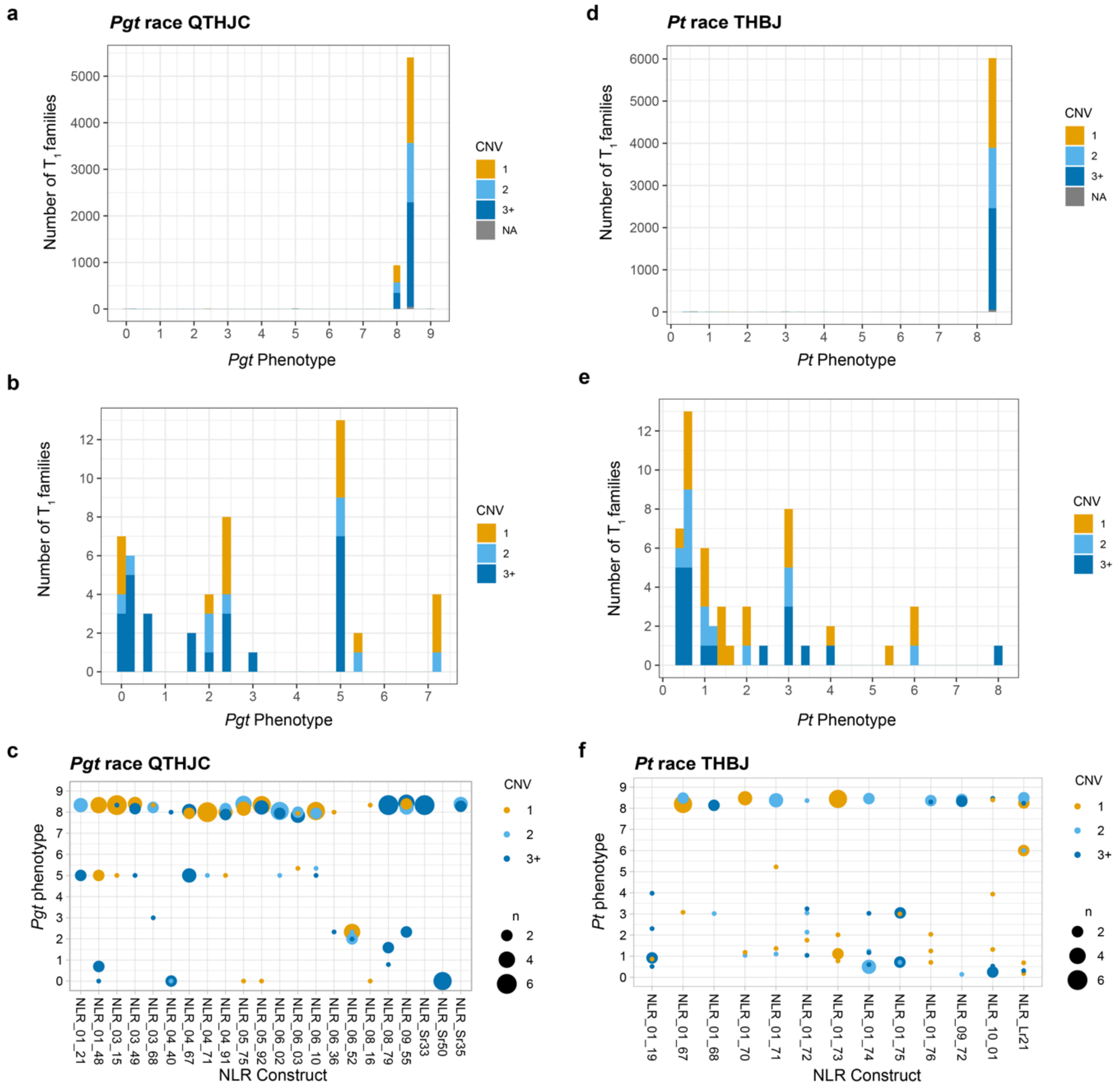

Supplemental Figure 7. Phenotypes of individuals from the 995 NLR array following inoculation with *Puccinia graminis* f. sp. *tritici* race QTHJC and *Puccinia triticina* race THBJ. Individual phenotypic scores for each T_1 transgenic line shown (**Supplemental Data 4**). CNV = Copy number variation. Copy number of individual T_1 lines indicated as 1 (orange), 2 (light blue), and 3 or more (dark blue) as determined by amplification of the hygromycin selectable marker. **A)** All individuals inoculated with *Pgt* race QTHJC showing most individuals with a susceptible score of < 8. **B)** Subset of the histogram for phenotypes less than 8 shown as an inset for clarity. **C)** Copy number of individual T_1 families per NLR construct resistant to *Pgt* race QTHJC. **D)** All individuals inoculated with *Pt* race THBJ showing most individuals with a susceptible score of <= 8. **E)** Subset of the histogram for phenotypes less than 8 shown as an inset for clarity. **F)** Copy number of individual T_1 families per NLR construct resistant to *Pt* race THBJ.

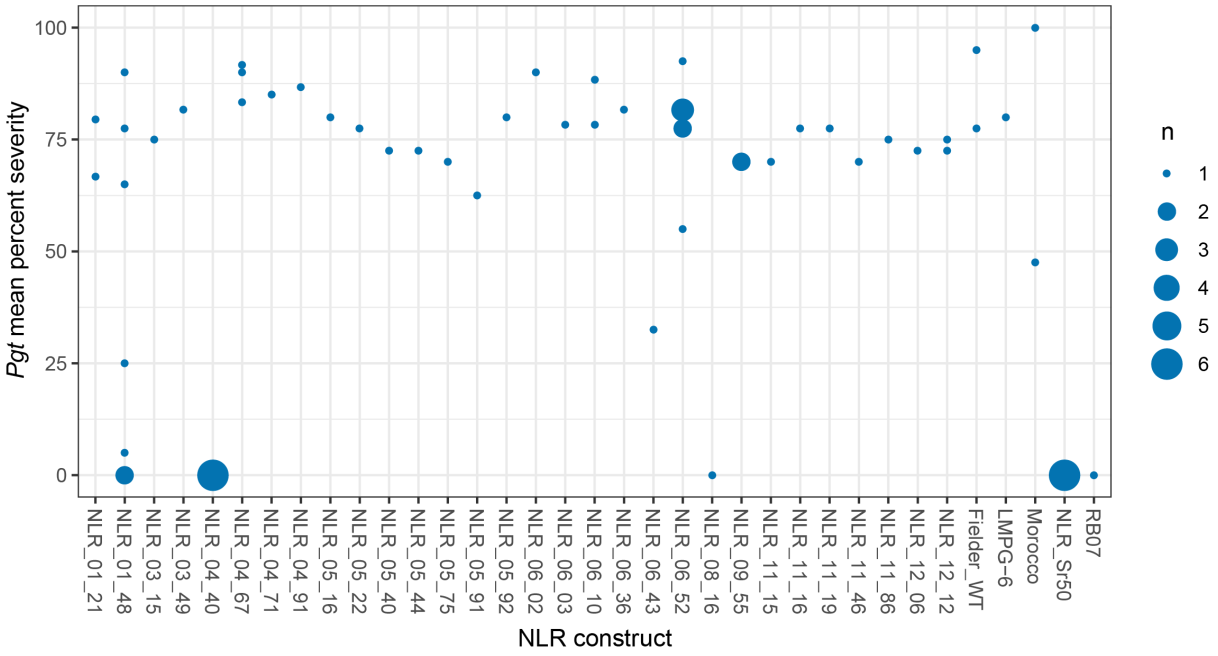

Supplemental Figure 8. Field resistance of adult plants to *Puccinia graminis* f. sp. *tritici* race QTHJC. Phenotypic scores from individuals within T_2 families from each construct inoculated with *Pgt* race QTHJC under field conditions during 2021 and 2022 (**Supplemental Data 5**). Circle size indicates number of individuals at each phenotypic score. Resistant controls included cultivar RB07 and transgenic lines with *Sr50*. The cultivars Fielder, Morocco, LMPG-6 were included as susceptible controls. Phenotypes scored as percent severity recorded as the visual percentage (0-100%) of tissue covered by uredinia. The lowest phenotype was used from segregating lines due to assumed segregation of the transgene. The average of the replicates per transgenic line is shown. Individuals from T_2 families of the constructs NLR_01_48, NLR_04_40, NLR_06_43, and NLR_08_16 displayed high levels of resistance.

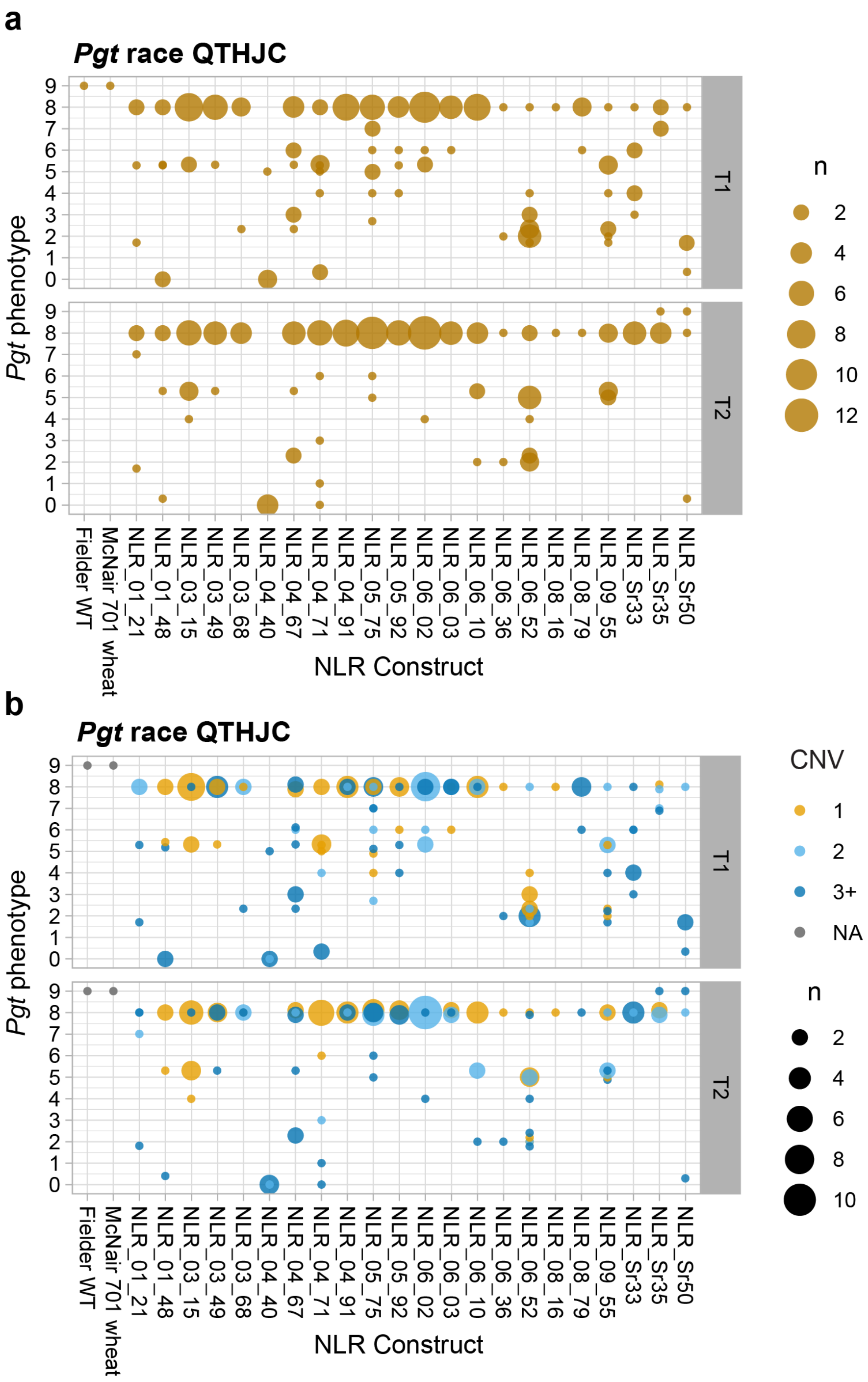

Supplemental Figure 9. Secondary screen of all independent T_1 and T_2 families per NLR construct conferring resistance to *Pgt*. A) Phenotypic scores from individuals within T_1 families from each construct inoculated with *Pgt* race QTHJC plotted on a weighted and transformed Stakman scale from highly resistant (0) to susceptible (9). Circle size indicates number of individuals having each phenotypic score. Susceptible controls Fielder wild-type and McNair 701 wheat. **B)** Replication of (A) with copy number of individual T_1 lines indicated as 1 (orange), 2 (light blue), and 3 or more (dark blue) as determined by amplification of the hygromycin selectable marker.

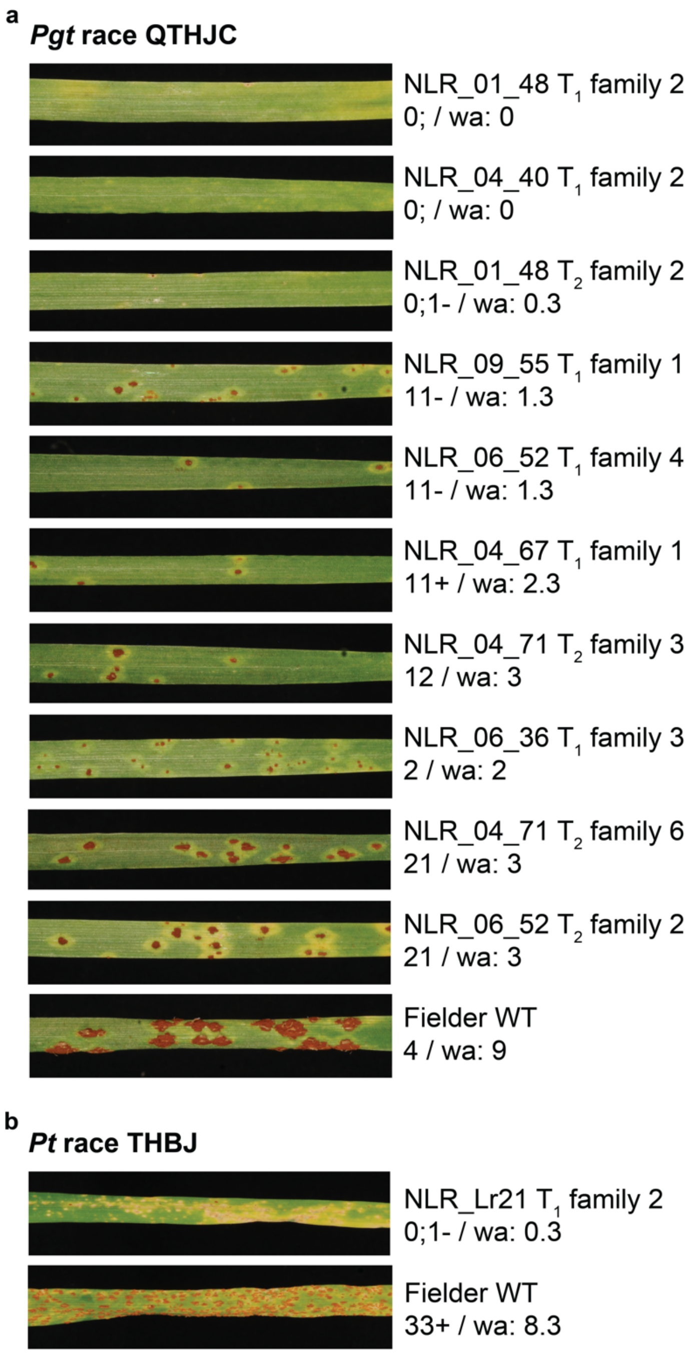

Supplemental Figure 10. Macroscopic phenotypes of *Puccinia graminis* f. sp. *tritici* (*Pgt*) race QTHJC and *Puccinia triticina* (*Pt*) race THBJ. Phenotypes shown as as infection types (IT) and the corresponding weighted average (wa). **A)** Seedling leaves of T_1 and T_2 individuals infected with *Pgt* race QTHJC under greenhouse conditions. Fielder wild type included as susceptible control. **B)** Seedling leaves of T_1 family of resistant control NLR_Lr21 and susceptible control Fielder wild type infected with *Pt* race THBJ.

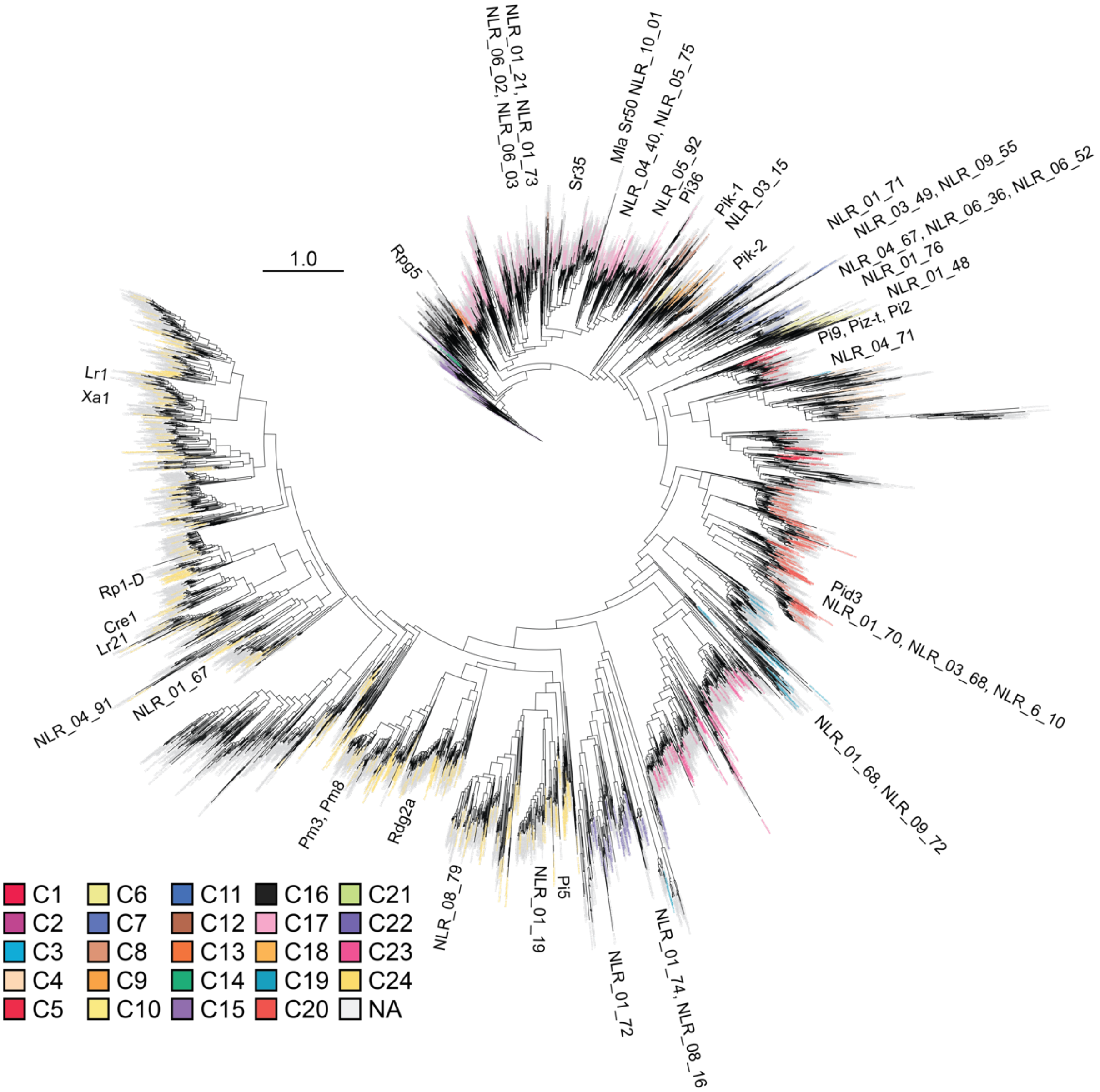

Supplemental Figure 11. Phylogenetic tree of diverse grass species NLR repertoire and resistant NLRs. The maximum likelihood phylogenetic tree is based on the NB domain of NLRs from *Aegilops tauschii*, *Brachypodium distachyon*, *Hordeum vulgare* (barley), *Oryza sativa* (rice), *Setaria italica*, *Sorghum bicolor*, *Triticum aestivum* (wheat), and *Triticum urartu*, *Zea mays* (maize), previously cloned NLRs, and resistant NLRs in this work using the approach and clade classification of Bailey *et al.* (2018). NLR identifiers are labelled based on clade classification (key in bottom right). Bootstrap support of greater than 80% is indicated by orange dots on branches based on 1,000 bootstraps. The phylogenetic tree contains 5,000 non-redundant NB domains from NLRs.

Supplemental Table 1. The number of accessions sequenced and the number of NLRs cloned and transformed per species.

Species	Acronym	Common name	Accessions	NLRs
Achnatherum hymenoides	*Ahy*	Indian ricegrass	3	20
Aegilops bicornis	*Abi*	Goat-hardgrass	6	95
Aegilops longissima	*Alo*	Long goatgrass	5	77
Aegilops searsii	*Aes*	Sears' goatgrass	3	52
Aegilops sharonensis	*Ash*	Sharon goatgrass	16	213
Agropyron cristatum	*Agc*	Crested Wheatgrass	2	31
Avena abyssinica	*Ava*	Abyssinian oat	3	51
Brachypodium distachyon	*Bdi*	Purple false brome	2	25
Briza media	*Brm*	Quaking grass	3	54
Cynosurus cristatus	*Ccr*	Crested dogtail grass	3	39
Echinaria capitata	*Ecc*	Hedgehog Grass	1	9
Holcus lanatus	*Hla*	Common velvet grass	3	46
Hordeum vulgare	*Hvu*	Barley	5	38
Koeleria macrantha	*Kma*	Prairie Junegrass	3	63
Lolium perenne	*Lop*	Perennial ryegrass	3	50
Melica ciliata	*Mci*	Hairy melic grass	2	50
Phalaris coerulescens	*Pco*	Blue canary grass	3	11
Poa trivialis	*Ptr*	Rough bluegrass	3	65

Supplemental Table 2. Resistant NLRs against the wheat stem rust (*Puccinia graminis* f. sp. *tritici; Pgt*) following screening of transgenic T_1 families with *Pgt* races QTHJC and TTKSK.

Construct	Species	Accession	Domain Structure	Clade	TPM	Number of resistant independent families	
						QTHJC	TTKSK[1]
NLR_01_21	*Aegilops sharonensis*	575	CC-NB-LRR	C17	4.93	2	1
NLR_01_48	*Aegilops sharonensis*	546	CC-NB-LRR	C6	2.00	4	2
NLR_03_15	*Cynosurus cristatus*	PI251810	NB-LRR	C12	3.56	1	0
NLR_03_49	*Holcus lanatus*	PI659841	NB-LRR	C7	2.00	1	0
NLR_03_68	*Aegilops sharonensis*	575	CC-NB-LRR	C20	2.42	1	0
NLR_04_40	*Aegilops longissima*	8735	CC-NB-LRR	C17	1.00	2	3
NLR_04_67	*Aegilops bicornis*	2327	CC-NB-LRR	C7	1.30	2	2
NLR_04_71	*Aegilops bicornis*	2327	CC-NB-LRR	C2	38.10	1	0
NLR_04_91	*Aegilops bicornis*	6065	CC-NB-LRR	C24	1.60	1	0
NLR_05_75	*Aegilops bicornis*	6065	CC-NB-LRR	C17	1.80	1	0
NLR_05_92	*Aegilops bicornis*	6150	NB-LRR	C17	1.00	1	0
NLR_06_02	*Aegilops longissima*	1059	CC-NB-LRR	C17	1.80	1	0
NLR_06_03	*Aegilops longissima*	1059	NB-LRR	C17	3.70	1	0
NLR_06_10	*Aegilops bicornis*	6150	CC-NB-LRR	C20	1.00	2	1
NLR_06_36	*Aegilops searsii*	6223	CC-NB-LRR	C7	1.96	1	1
NLR_06_52	*Aegilops searsii*	6229	CC-NB-LRR	C7	1.28	4	3
NLR_08_16	*Aegilops sharonensis*	2020	RPW8-NB-LRR	C22	27.13	1	0
NLR_08_79	*Avena abyssinica*	PI158202	CC-NB-LRR-CC-LRR	C24	5.19	1	1
NLR_09_55	*Briza media*	PI3212443	CC-NB-LRR	C7	2.78	1	1

Supplemental Table 3. Summary of secondary screen of resistant NLRs against the wheat stem rust (*Puccinia graminis* f. sp. *tritici; Pgt*) of transgenic T_1 and T_2 families with *Pgt* race QTHJC.

Construct	Species	Accession	Domain Structure	Clade	TPM	T1 Families			T2 Families		
						Resistant	Total	%	Resistant	Total	%
NLR_01_21	*Aegilops sharonensis*	575	CC-NB-LRR	C17	4.93	2	3	66.7	1	3	33.3
NLR_01_48	*Aegilops sharonensis*	546	CC-NB-LRR	C6	2	4	5	80	2	3	66.7
NLR_03_15	*Cynosurus cristatus*	PI251810	NB-LRR	C12	3.56	1	7	14.3	2	7	28.6
NLR_03_49	*Holcus lanatus*	PI659841	NB-LRR	C7	2	1	5	20	1	4	25
NLR_03_68	*Aegilops sharonensis*	575	CC-NB-LRR	C20	2.42	1	3	33.3	0	3	0
NLR_04_40	*Aegilops longissima*	8735	CC-NB-LRR	C17	1	3	3	100	3	3	100
NLR_04_67	*Aegilops bicornis*	2327	CC-NB-LRR	C7	1.3	5	6	83.3	2	5	40
NLR_04_71	*Aegilops bicornis*	2327	CC-NB-LRR	C2	38.1	7	7	100	4	7	57.1
NLR_04_91	*Aegilops bicornis*	6065	CC-NB-LRR	C24	1.6	0	4	0	0	4	0
NLR_05_75	*Aegilops bicornis*	6065	CC-NB-LRR	C17	1.8	4	7	57.1	2	7	28.6
NLR_05_92	*Aegilops bicornis*	6150	NB-LRR	C17	1	2	4	50	0	3	0
NLR_06_02	*Aegilops longissima*	1059	CC-NB-LRR	C17	1.8	2	7	28.6	1	7	14.3
NLR_06_03	*Aegilops longissima*	1059	NB-LRR	C17	3.7	1	4	25	0	3	0
NLR_06_10	*Aegilops bicornis*	6150	CC-NB-LRR	C20	1	0	4	0	2	4	50
NLR_06_36	*Aegilops searsii*	6223	CC-NB-LRR	C7	1.96	1	2	50	1	2	50
NLR_06_52	*Aegilops searsii*	6229	CC-NB-LRR	C7	1.28	7	7	100	7	7	100
NLR_08_16	*Aegilops sharonensis*	2020	RPW8-NB-LRR	C22	27.13	0	1	0	0	1	0
NLR_08_79	*Avena abyssinica*	PI158202	CC-NB-LRR-CC-LRR	C24	5.19	1	4	25	0	1	0
NLR_09_55	*Briza media*	PI3212443	CC-NB-LRR	C7	2.78	6	7	85.7	4	6	66.7
NLR_Sr33	*Aegilops tauschii*	RL5288	-	-	-	4	5	80	0	5	0
NLR_Sr50	*Secale cereale*	Imperial	-	-	-	3	4	75	1	3	33.3
NLR_Sr35	*Triticum monococcum*	PI428170	-	-	-	0	4	0	0	5	0

Supplemental Table 4. Resistant NLRs against the wheat leaf rust (*Puccinia triticina; Pt*) following screening of transgenic T_1 families with *Pt* races THBJ.

**Construct**	**Species**	**Accession**	**Domain Structure**	**Clade**	**TPM**	**Number of resistant independent families to THBJ**
NLR_01_19	*Aegilops sharonensis*	546	CC-NB-LRR	C24	15.65	4
NLR_01_67	*Cynosurus cristatus*	PI642806	NB-LRR	C24	0.98	1
NLR_01_68	*Cynosurus cristatus*	PI642806	NB-LRR	C19	0.94	1
NLR_01_70	*Cynosurus cristatus*	PI642806	NB-LRR	C20	0.85	2
NLR_01_71	*Cynosurus cristatus*	PI642806	NB-LRR	C7	1.80	3
NLR_01_72	*Holcus lanatus*	PI325350	NB-LRR	C22	5.20	4
NLR_01_73	*Holcus lanatus*	PI325350	NB-LRR	C17	6.81	3
NLR_01_74	*Holcus lanatus*	PI325350	CC-NB-LRR	C22	30.09	3
NLR_01_75	*Holcus lanatus*	PI325350	NB-LRR	C20	4.43	4
NLR_01_76	*Holcus lanatus*	PI325350	NB-LRR	C7	1.65	2
NLR_09_72	*Echinaria capitata*	PI657658	CC-NB-LRR	C19	3.88	1
NLR_10_01	*Hordeum vulgare*	Heils Franken	CC-NB-LRR	C17	43.98	4
