# Supplementary material for: Discovery of functional NLRs using expression level, high-throughput transformation and large-scale phenotyping
Source: Nat Plants. 2025 Sep 23;11(10):2100–14. doi: 10.1038/s41477-025-02110-w (PMC12537499; doi:10.1038/s41477-025-02110-w)
Supplement: Supplementary file 2 — Reporting Summary [file 41477_2025_2110_MOESM2_ESM.pdf]

nature portfolio

Corresponding author(s): Helen Brabham

Last updated by author(s): 06/02/2025

Reporting Summary

Nature Portfolio wishes to improve the reproducibility of the work that we publish. This form provides structure for consistency and transparency in reporting. For further information on Nature Portfolio policies, see our Editorial Policies and the Editorial Policy Checklist.

Statistics

For all statistical analyses, confirm that the following items are present in the figure legend, table legend, main text, or Methods section.

n/a	Confirmed	
☐	☒	The exact sample size (*n*) for each experimental group/condition, given as a discrete number and unit of measurement
☐	☒	A statement on whether measurements were taken from distinct samples or whether the same sample was measured repeatedly
☒	☐	The statistical test(s) used AND whether they are one- or two-sided<br>*Only common tests should be described solely by name; describe more complex techniques in the Methods section.*
☒	☐	A description of all covariates tested
☒	☐	A description of any assumptions or corrections, such as tests of normality and adjustment for multiple comparisons
☐	☒	A full description of the statistical parameters including central tendency (e.g. means) or other basic estimates (e.g. regression coefficient) AND variation (e.g. standard deviation) or associated estimates of uncertainty (e.g. confidence intervals)
☒	☐	For null hypothesis testing, the test statistic (e.g. *F*, *t*, *r*) with confidence intervals, effect sizes, degrees of freedom and *P* value noted<br>*Give P values as exact values whenever suitable.*
☒	☐	For Bayesian analysis, information on the choice of priors and Markov chain Monte Carlo settings
☒	☐	For hierarchical and complex designs, identification of the appropriate level for tests and full reporting of outcomes
☒	☐	Estimates of effect sizes (e.g. Cohen's *d*, Pearson's *r*), indicating how they were calculated

Our web collection on statistics for biologists contains articles on many of the points above.

Software and code

Policy information about availability of computer code

Data collection	Full descriptions of bioinformatics software and versions are listed in the materials and methods.
Data analysis	Software packages and tools are listed in the materials and methods:<br>FastQC (0.11.7)<br>Trimmomatic (v0.39)<br>k-mer analysis toolkit (KAT; v2.4.1)<br>R (v4.1.2)<br>ggplot2 (v3.3.6)<br>Trinity (version 2013-11-10)<br>kallisto (v0.43.1)<br>BLAST (v2.2.31)<br>Geneious Prime (v2024.0.3)<br>Fiji (ImageJ2, v2.14.0/1.5f)<br>RStudio (v2023.12.1+402)<br>TransDecoder (v4.1.0)<br>InterProScan (v5.27-66.0)<br>MUSCLE (v5.1)<br>Fast Approximate Tree Classification (FAT-CAT)<br>CD-HIT (v4.7)<br>HMMer (v3.3.2)<br>QKphylogeny scripts (https://github.com/matthewmoscou/QKphylogeny)

RAxML (v8.2.12)
iTOL (https://itol.embl.de/)
QKbusco pipeline (https://github.com/matthewmoscou/QKbusco)
BUSCO (v3.0.2)
TransDecoder (v4.1.0)
PRANK (v.170427)

For manuscripts utilizing custom algorithms or software that are central to the research but not yet described in published literature, software must be made available to editors and reviewers. We strongly encourage code deposition in a community repository (e.g. GitHub). See the Nature Portfolio guidelines for submitting code & software for further information.

Data

Policy information about availability of data

All manuscripts must include a data availability statement. This statement should provide the following information, where applicable:

- Accession codes, unique identifiers, or web links for publicly available datasets
- A description of any restrictions on data availability
- For clinical datasets or third party data, please ensure that the statement adheres to our policy

Whole genome sequencing data of barley accessions CI 16147 and CI 16153 were deposited in NCBI BioProject PRJNA952654. RNAseq data for Arabidopsis thaliana, tomato, and diverse Poodieae species were deposited in NCBI BioProject PRJNA928100, PRJNA927036, and PRJNA913397, respectively. GenBank identifiers for transformation construct sequence for Mla7 under Mla6 promoter/terminator and native sequence are MZ555770 and OQ859100, respectively. Databases used for protein domain analysis include Pfam, Superfamily, and ProSite. Raw data, uncropped images, and scripts used for data analysis and figure preparation were deposited on Figshare (https://doi.org/10.1101/2024.06.25.599845; https://doi.org/10.1101/2024.06.25.599845).

Research involving human participants, their data, or biological material

Policy information about studies with human participants or human data. See also policy information about sex, gender (identity/presentation), and sexual orientation and race, ethnicity and racism.

Reporting on sex and gender	na
Reporting on race, ethnicity, or other socially relevant groupings	na
Population characteristics	na
Recruitment	na
Ethics oversight	na

Note that full information on the approval of the study protocol must also be provided in the manuscript.

Field-specific reporting

Please select the one below that is the best fit for your research. If you are not sure, read the appropriate sections before making your selection.

☒ Life sciences ☐ Behavioural & social sciences ☐ Ecological, evolutionary & environmental sciences

For a reference copy of the document with all sections, see nature.com/documents/nr-reporting-summary-flat.pdf

Life sciences study design

All studies must disclose on these points even when the disclosure is negative.

Sample size	Sample size details are listed in the materials and methods. For plant inoculations, sample sizes were used that are standard in the field with 3 biological replicates per sample. Sample sizes of independent transgenic events ranged from 2 to 7 independent events per transgene, with 3 plants sampled per independent event due to the breadth of the study.
Data exclusions	No data were excluded from the analyses.
Replication	Experimental replication is detailed in the materials and methods. Plant phenotyping experiments were performed with 3 biological replicates per sample. All replication attempts were successful and no subsampling of results have been performed in this manuscript. For the Phytophthora infestans inoculations 3 to 4 biological replicates were performed within technical replicates.
Randomization	Non-randomization, does not apply to plant inoculations as plants were grown and treated with the same inocculum. All experiments were inherently randomly performed as there is no specific structure to the collection of NLR candidate genes.

Blinding	Semi-blinding was performed as plant were phenotyped and data recorded based on the plant ID number without gene information.

Reporting for specific materials, systems and methods

We require information from authors about some types of materials, experimental systems and methods used in many studies. Here, indicate whether each material, system or method listed is relevant to your study. If you are not sure if a list item applies to your research, read the appropriate section before selecting a response.

Materials & experimental systems

n/a	Involved in the study	
☒	☐	Antibodies
☒	☐	Eukaryotic cell lines
☒	☐	Palaeontology and archaeology
☒	☐	Animals and other organisms
☒	☐	Clinical data
☒	☐	Dual use research of concern
☐	☒	Plants

Methods

n/a	Involved in the study	
☒	☐	ChIP-seq
☒	☐	Flow cytometry
☒	☐	MRI-based neuroimaging

Dual use research of concern

Policy information about dual use research of concern

Hazards

Could the accidental, deliberate or reckless misuse of agents or technologies generated in the work, or the application of information presented in the manuscript, pose a threat to:

No	Yes	
☒	☐	Public health
☒	☐	National security
☒	☐	Crops and/or livestock
☒	☐	Ecosystems
☒	☐	Any other significant area

Experiments of concern

Does the work involve any of these experiments of concern:

No	Yes	
☒	☐	Demonstrate how to render a vaccine ineffective
☒	☐	Confer resistance to therapeutically useful antibiotics or antiviral agents
☒	☐	Enhance the virulence of a pathogen or render a nonpathogen virulent
☒	☐	Increase transmissibility of a pathogen
☒	☐	Alter the host range of a pathogen
☒	☐	Enable evasion of diagnostic/detection modalities
☒	☐	Enable the weaponization of a biological agent or toxin
☒	☐	Any other potentially harmful combination of experiments and agents

Plants

Seed stocks	Seeds were obtained from The C.M. Rick Tomato Genetics Resource Center (TGRC; https://tgrc.ucdavis.edu/) and the Nottingham Arabidopsis Stock Centre (https://arabidopsis.info/). Seed stocks are listed in the materials and methods.
Novel plant genotypes	Transgenic lines were generated through Agrobacterium-mediated transformation of the wheat cultivar Fielder (Ishida et al., 2015). Genotyping was performed on the T0 and pathogen inoculations performed on the T1 and subsequent T2 generations.
Authentication	Transgenic lines were validated through genotyping of the selectable marker.
